# The role of OTUD1-mediated deubiquitination in disease pathogenesis: from molecular mechanisms to clinical translation

**DOI:** 10.3389/fimmu.2026.1877213

**Published:** 2026-07-22

**Authors:** Qingsong Wang, LiJuan Zhang, Hongmei Cao, Xianmin Wang, Tongyong Luo, Jun Yin

**Affiliations:** 1 Department of Pediatrics, West China Hospital Sichuan University Jintang Hospital, Jintang First People’s Hospital, Chengdu, Sichuan, China; 2 Department of Surgery, West China Hospital Sichuan University Jintang Hospital, Jintang First People’s Hospital, Chengdu, Sichuan, China; 3 Department of Obstetrics and Gynecology, West China Hospital Sichuan University Jintang Hospital, Jintang First People’s Hospital, Chengdu, Sichuan, China; 4 Pediatric Cardiology Center, Sichuan Provincial Women’s and Children’s Hospital/The Affiliated Women’s and Children’s Hospital of Chengdu Medical College, Chengdu, Sichuan, China; 5 Department of Ultrasound, West China Hospital Sichuan University Jintang Hospital, Jintang First People’s Hospital, Chengdu, Sichuan, China

**Keywords:** biomarker, deubiquitinase, disease mechanism, OTUD1, targeted therapy, tumor, ubiquitin regulation

## Abstract

OTUD1 is a deubiquitinase of the OTU family that participates in immune signaling, redox balance, and cell death through modulating substrate ubiquitination. Under physiological conditions, OTUD1 primarily exerts negative regulatory effects, restricting excessive activation of NF-κB inflammatory signaling and type I interferon responses, thereby maintaining immune and oxidative homeostasis. Under pathological conditions, its functional effects undergo tissue-dependent reprogramming: it is downregulated in most epithelial tumors and suppresses malignant progression, whereas it is upregulated in certain tumors and cardiovascular or metabolic diseases and promotes pathological remodeling. It exerts protective regulation in the nervous system and mucosal inflammation, yet drives hypertrophy and fibrosis in the cardiovascular system. Preclinical studies indicate that OTUD1 expression levels correlate with tumor stage, prognosis, and therapeutic sensitivity, suggesting potential as a biomarker for diagnostic subtyping and prognostic assessment. However, marked tissue functional heterogeneity, the discovery of non-enzymatic scaffold functions, and the lack of highly selective targeting probes constitute central bottlenecks for clinical translation. This review systematically summarizes the molecular characteristics, physiological functions, disease regulatory networks, and current clinical translational status of OTUD1, with the aim of clarifying the molecular basis of its functional duality and providing a theoretical foundation for optimizing precision diagnostic and therapeutic strategies targeting OTUD1.

## 1 Biological characteristics of OTUD1

### 1.1 Molecular features

OTUD1 (ovarian tumor domain-containing protein 1, also known as DUBA7 or OTDC1) is a member of the ovarian tumor protease (OTU) family of deubiquitinases encoded by a single-exon gene located on human chromosome 10p12.2 (1). Its gene product comprises 481 amino acid residues with a molecular weight of approximately 51 kDa (2, 3).

**Figure 1:**
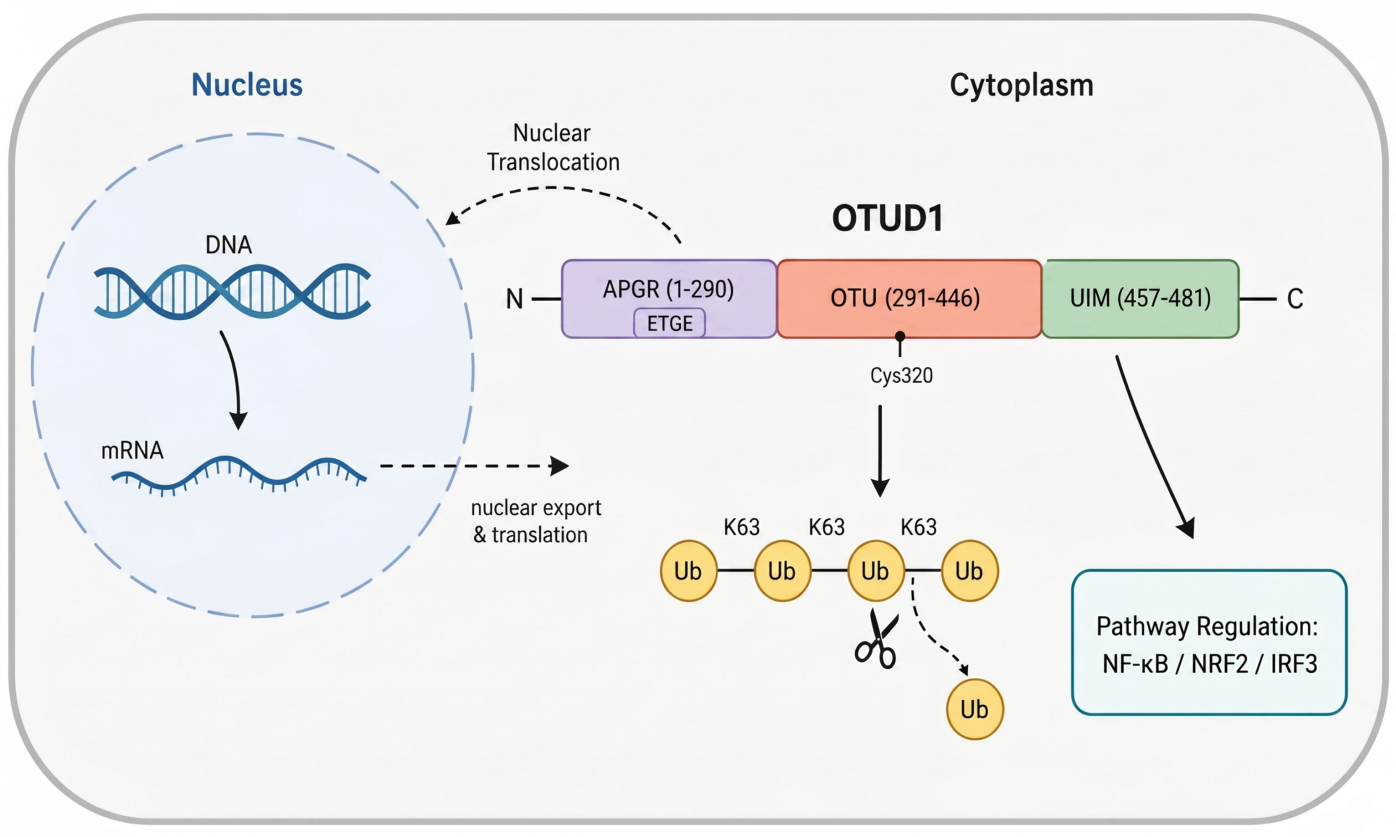
Molecular structure and deubiquitinating mechanism of OTUD1. OTUD1 is a member of the ovarian tumor (OTU) family of deubiquitinating enzymes (DUBs). **(A)** Structural domains: OTUD1 protein consists of an N-terminal APGR domain (aa 1–290) containing an ETGE motif, a central catalytic OTU domain (aa 291–446) with the active site Cys320, and a C-terminal Ubiquitin Interacting Motif (UIM, aa 457–481). **(B)** Expression and localization: Following gene transcription in the nucleus and mRNA export, OTUD1 is translated in the cytoplasm. Under specific stimuli, OTUD1 can undergo nuclear translocation to regulate nuclear substrates. © Enzymatic function: OTUD1 specifically recognizes and cleaves K63-linked polyubiquitin chains through its OTU and UIM domains, thereby regulating multiple downstream signaling pathways, including NF-κB, NRF2, and IRF3.

At the protein structural level, OTUD1 consists of three main regions (**Figure 1**): an N-terminal intrinsically disordered Ala-, Pro-, and Gly-rich region (APGR, amino acids 1–290), a centrally located catalytically active OTU domain (amino acids 291–446), and a C-terminal ubiquitin-interacting motif (UIM, amino acids 457–481) (2, 4). The catalytic activity of OTUD1 depends on the conserved cysteine residue Cys320 within the OTU domain; mutation to serine (C320S) completely abolishes its deubiquitinase activity (3, 5). The APGR region is predicted by bioinformatics to be an intrinsically disordered low-complexity domain, with an exceptionally high content of alanine (21.7%), proline (16.9%), and glycine (9.6%). Within this region lies an evolutionarily conserved ETGE motif (ETGE in humans, DTGE in mice, amino acids 49–52), which serves as the critical binding site for direct interaction with the KEAP1 protein (4).

Regarding substrate specificity, *in vitro* enzymatic assays demonstrate that full-length OTUD1 and OTU+UIM constructs exhibit high hydrolytic activity and specificity toward K63-linked ubiquitin chains. In contrast, the isolated OTU domain shows significantly reduced activity and loss of substrate specificity, suggesting that the C-terminal UIM domain is essential for efficient recognition and cleavage of K63-linked chains by OTUD1 (6). Although K63 chains are the preferred substrate *in vitro*, OTUD1 also participates in hydrolyzing K6-, K11-, K27-, K29-, K33-, and K48-linked ubiquitin chains in cells (2, 7, 8).

With respect to subcellular localization, OTUD1 primarily functions in the cytoplasm. Under specific stimulatory conditions, it interacts with substrate proteins (such as STAT3 and IRF3) and undergoes nuclear translocation concurrently (5, 7). OTUD1 is basally expressed in various tissue cells, including cardiomyocytes, vascular endothelial cells, renal tubular epithelial cells, and macrophages (5, 9, 10).

### 1.2 Expression regulation mechanisms

The expression of OTUD1 is subject to multi-layered regulation, encompassing transcriptional control, epigenetic modification, and post-transcriptional mRNA regulation.

At the transcriptional level, multiple extracellular stimuli can induce upregulation of OTUD1. Under inflammatory stimulation, lipopolysaccharide (LPS), polyinosinic:polycytidylic acid [poly(I:C)], Sendai virus (SeV), and pro-inflammatory cytokines TNF-α and IL-1β significantly increase OTUD1 mRNA and protein levels (4, 7). The transcription factor FOXO3 plays a key role in this process. Studies have shown that AKT signaling inhibition caused by serum starvation or viral infection promotes FOXO3 translocation from the cytoplasm to the nucleus; nuclear FOXO3 directly binds to conserved sequences in the OTUD1 promoter to initiate transcription (7). Knockdown of FOXO3 reduces virus-induced OTUD1 upregulation by approximately 80% (Sendai virus-infected HEK293 cells, *in vitro*, qRT-PCR and western blot) (7).

At the post-transcriptional mRNA regulation level, N6-methyladenosine (m6A) modification represents another important mechanism controlling OTUD1 expression, with diametrically opposite effects mediated by different “reader” proteins in distinct physiological and pathological contexts. In the heart, METTL3-mediated m6A methylation enhances OTUD1 mRNA stability; YTHDF1, acting as a reader protein, specifically recognizes this modification site and promotes OTUD1 protein translation efficiency (11). However, in airway epithelial cells, m6A modifications catalyzed by the same METTL3 are recognized by another reader protein, YTHDF2, which accelerates OTUD1 mRNA degradation and reduces its protein level (1). Additionally, DNA methylation can silence OTUD1 transcription. In non-small cell lung cancer and other malignancies, hypermethylation of CpG islands in the OTUD1 gene promoter region is an important mechanism underlying its downregulation (12).

### 1.3 Physiological functions

**Figure 2:**
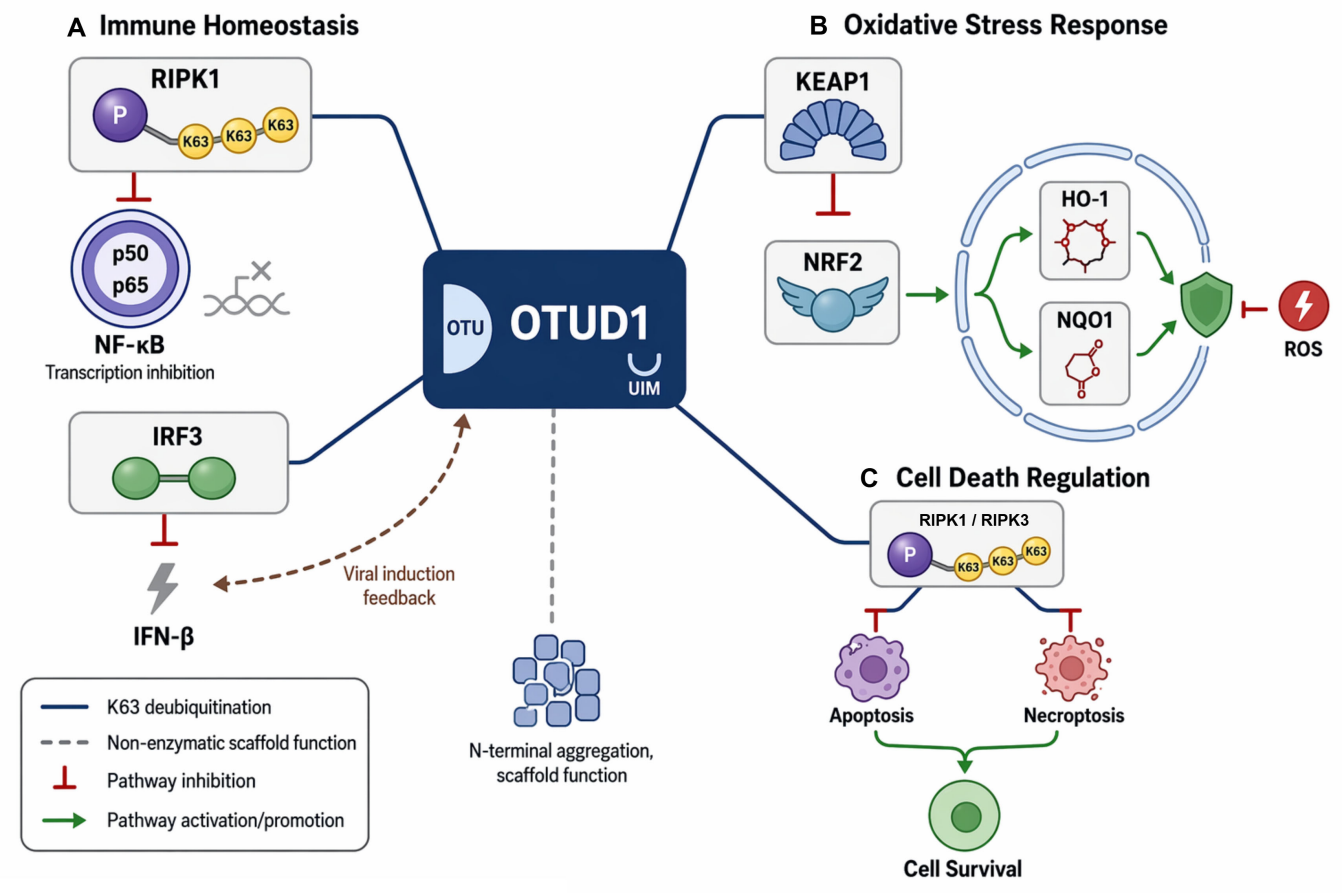
Physiological functions of OTUD1 in maintaining cellular homeostasis. **(A)** In immune homeostasis, OTUD1 removes K63-linked ubiquitin chains from RIPK1 to restrict NF-κB activation, and deubiquitinates IRF3 to suppress IFN-β overproduction, forming a viral-inducible negative feedback loop. **(B)** In oxidative stress response, OTUD1 engages KEAP1 via its N-terminal ETGE motif and promotes NRF2 nuclear accumulation, driving antioxidant gene transcription (HO-1 and NQO1) to neutralize ROS. **(C)** In cell death regulation, OTUD1 hydrolyzes K63-linked ubiquitin chains from RIPK1 and RIPK3 to block apoptosis and necroptosis, thereby promoting cell survival; the N-terminal intrinsically disordered region also mediates aggresome formation, exerting a non-enzymatic scaffold function independent of Cys320 catalytic activity.

Under normal physiological conditions, OTUD1 regulates multiple signaling pathways primarily through its deubiquitinase activity, playing a key role in maintaining immune homeostasis, redox balance, and cell fate determination (**Figure 2**).

#### 1.3.1 Inflammation and immune regulation

OTUD1 is an important regulator for maintaining immune homeostasis. In the canonical NF-κB signaling pathway, OTUD1 exerts negative regulatory functions. When cells are stimulated by pro-inflammatory signals such as TNF-α or IL-1β, OTUD1 is recruited to the corresponding receptor signaling complexes, where it hydrolyzes K63-linked polyubiquitin chains on RIPK1, NEMO (IKKγ), IRAK1, and LUBAC subunits to restrict IKK kinase complex activation and prevent excessive NF-κB signaling (4). Domain analysis indicates that OTUD1 binds directly to the kinase domain of RIPK1 via its N-terminal APGR region and specifically removes K63-linked ubiquitin chains at lysine 627 (K627) of RIPK1, thereby inhibiting NEMO recruitment to RIPK1 and limiting excessive NF-κB activation (13).

In the type I interferon antiviral response, OTUD1 similarly implements negative feedback regulation to maintain immune homeostasis. When cells are infected by viruses, the induced OTUD1 binds to the proline-rich region of transcription factor IRF3 via its APGR region and catalyzes removal of K63-linked ubiquitin chains at lysine 98 (K98) of IRF3 (7). This deubiquitination modification inhibits IRF3 dimerization, nuclear translocation, and transcriptional activity, thereby imposing negative feedback inhibition on IFN-β production to prevent tissue damage from excessive immune responses.

#### 1.3.2 Oxidative stress

Otud1 is a component of the intracellular antioxidant defense network. Under physiological conditions, cells continuously produce low levels of reactive oxygen species (ROS), and OTUD1 participates in fine regulation of redox balance through direct interaction with KEAP1. Specifically, OTUD1 utilizes the ETGE motif within its N-terminal APGR region to bind the C-terminal Kelch domain of KEAP1 (4). KEAP1 serves as the substrate adaptor for the Cullin 3 (CUL3) E3 ubiquitin ligase complex, normally mediating ubiquitination of transcription factor NRF2 and its subsequent proteasomal degradation. OTUD1 can remove K63-linked ubiquitin chains from KEAP1, modulating KEAP1 functional status and thereby indirectly affecting basal transcription levels of NRF2 downstream antioxidant genes (such as HO-1 and NQO1) (4). This regulatory mechanism constitutes a fundamental line of defense for cellular response to oxidative stress and is essential for maintaining intracellular redox homeostasis.

#### 1.3.3 Cell death

OTUD1 is an important node in regulating cell death signal transduction. In TNF-α-mediated cell death signaling pathways, OTUD1 exerts inhibitory effects on apoptosis and necroptosis, involving regulation of the ubiquitination status of multiple key signaling molecules. When OTUD1 functions normally, it effectively removes K63-linked ubiquitin chains from RIPK1 and RIPK3, thereby limiting assembly of downstream death signaling complexes: it inhibits formation of complex II composed of FADD and caspase-8 to block apoptosis, while simultaneously suppressing activation of the RIPK3-MLKL necrosome to block necroptosis (4). Thus, OTUD1 is an important regulatory node for cell survival signals, fine-tuning ubiquitination signals to regulate cell survival or death decisions under stress and injury.

Furthermore, OTUD1 possesses physiological functions independent of its deubiquitinase activity. Its N-terminal intrinsically disordered region (amino acids 105–164) can mediate supramolecular self-assembly of OTUD1 protein, forming cytoplasmic aggresomes in cells. This aggregation behavior is independent of Cys320 catalytic activity and is regulated by the microtubule system, suggesting that OTUD1 may function as a scaffold protein participating in protein homeostasis maintenance and signaling complex assembly (14).

## 2 OTUD1 in pathophysiological processes: multidimensional regulatory mechanisms

### 2.1 Immune homeostasis and inflammatory regulation

#### 2.1.1 Ulcerative colitis

The pathogenesis of ulcerative colitis is associated with genetic susceptibility, intestinal dysbiosis, barrier dysfunction, and abnormal immune responses; excessive activation of NF-κB and other signaling pathways drives pro-inflammatory cytokine release and tissue damage (15, 16). In dextran sulfate sodium-induced colitis mouse models, OTUD1 expression is compensatorily upregulated, and its functional loss exacerbates intestinal inflammation (13). OTUD1 binds to the RIPK1 kinase domain via its N-terminal Ala-rich region, removes K63-linked polyubiquitin chains at lysine 627 of RIPK1, inhibits NEMO recruitment to RIPK1, and thereby blocks NF-κB pathway activation and downstream pro-inflammatory cytokine transcription (4, 13). The ulcerative colitis-associated G430V mutant completely loses its deubiquitinating activity toward RIPK1 and its NF-κB inhibitory function; the identification of this mutation in patients directly links OTUD1 loss-of-function mutations to inflammatory bowel disease pathogenesis (13).

#### 2.1.2 Sepsis

Sepsis-induced acute lung injury is characterized by excessive inflammatory responses and alveolar barrier disruption, with Toll-like receptor 4-mediated inflammatory signals participating in its pathogenesis (17, 18). In peripheral blood from septic patients and in cecal ligation and puncture-induced sepsis mouse models, OTUD1 expression is upregulated and exerts immunoregulatory effects (19). OTUD1 directly interacts with TIPE2, removes K63-linked ubiquitin chains from TIPE2 through C320 catalytic activity, and stabilizes TIPE2 protein; stabilized TIPE2 inhibits TAK1 phosphorylation, blocks downstream MAPK and NF-κB signal pathway activation, and alleviates pulmonary inflammation and injury (19).

#### 2.1.3 Chronic obstructive pulmonary disease

Chronic obstructive pulmonary disease (COPD) is characterized by persistent airflow limitation and chronic airway inflammation; smoking is the major risk factor, and cigarette smoke extract can induce inflammation, pyroptosis, and epithelial-mesenchymal transition in airway epithelial cells (20–25). In advanced COPD patient lung tissue and in cigarette smoke extract-stimulated airway epithelial cells, OTUD1 expression is downregulated, weakening its inhibitory effects on inflammation and pyroptosis (1). Cigarette smoke extract accelerates OTUD1 mRNA degradation through METTL3/YTHDF2-mediated m6A methylation, leading to decreased protein levels; insufficient OTUD1 fails to restrain NF-κB/NLRP3/GSDMD-mediated pyroptosis and inflammation, accelerating disease progression. Knockdown of METTL3 or YTHDF2 restores OTUD1 expression and alleviates cigarette smoke extract-induced inflammation and pyroptosis (1).

#### 2.1.4 Autoimmune diseases

Autoimmune diseases are characterized by abnormal immune responses against self-antigens causing tissue damage; excessive activation of the type I interferon pathway and downstream interferon-stimulated gene transcription are associated with the pathogenesis of multiple autoimmune diseases (26). Lu et al. performed exon sequencing of OTUD1 in 306 autoimmune disease patients and 300 healthy subjects (China-Japan Friendship Hospital cohort, SLE/RA/UC/HT, in human, exon sequencing) (7). Wild-type OTUD1 serves as a negative regulator of RIG-I-like receptor pathways and type I interferon synthesis, and viral stimulation upregulates its expression. OTUD1 binds to the proline-rich region of transcription factor IRF3 via its APGR domain, removes K63-type ubiquitin chains at lysine 98 of IRF3, blocks IRF3 nuclear translocation, and inhibits its transcriptional activity, thereby negatively regulating IFN-β secretion (7). The identified pathogenic OTUD1 mutations impair IRF3 deubiquitination to varying degrees, inducing aberrant hyperactivation of RIG-I-like receptor pathways and type I interferon responses. The P95R mutation does not affect enzymatic activity, but because the mutation site is located in the APGR domain, it disrupts the interaction between OTUD1 and IRF3, leading to loss of immunoregulatory function (7). In inflammatory microenvironments, the transcription factor FOXO3 is the upstream molecule mediating OTUD1-induced expression.

#### 2.1.5 Hypertensive kidney injury

Hypertensive kidney injury is a common cause of chronic kidney disease and end-stage renal disease, with pathological features including renal tubular epithelial cell injury, inflammatory cell infiltration, and renal interstitial fibrosis; angiotensin II (Ang II)participates in its pathogenesis (27, 28). In angiotensin II-infused hypertensive mouse model renal tubular epithelial cells, OTUD1 expression is elevated and exerts pro-pathological effects (10). OTUD1 directly binds to CDK9, removes K63-linked ubiquitin chains from CDK9, promotes CDK9 phosphorylation and increased kinase activity; activated CDK9 subsequently activates the NF-κB signaling pathway and expression of inflammatory factors including TNF-α, IL-6, and IL-1β, driving renal inflammation and fibrosis. CDK9 selective inhibitors can reverse OTUD1 overexpression-induced kidney injury (10).

#### 2.1.6 Heart failure and myocardial infarction

Heart failure and myocardial infarction are associated with chronic sympathetic overactivation, which can trigger cardiomyocyte hypertrophy, apoptosis, and interstitial fibrosis (3). In isoproterenol, myocardial infarction, and pressure overload-induced heart failure models, OTUD1 expression is upregulated in macrophages, indirectly driving cardiac remodeling through inflammatory signals (29). OTUD1 removes K33-linked ubiquitin chains from CARD9, promoting CARD9 assembly with BCL10 and MALT1 into the CBM signaling complex, thereby activating the NF-κB signaling pathway; activated macrophages secrete inflammatory factors that induce cardiomyocyte hypertrophy and fibroblast activation (29).

**Figure 3:**
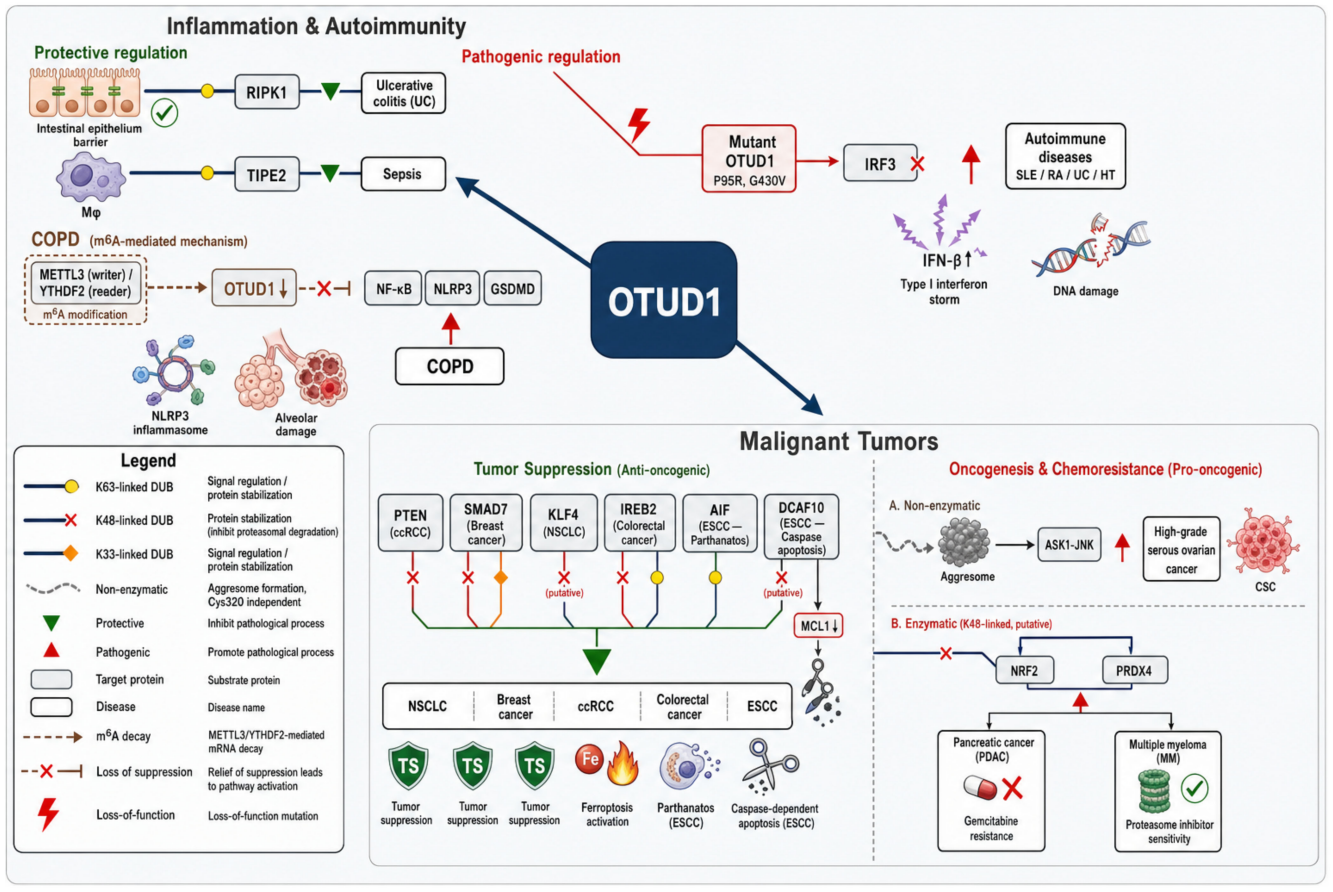
Regulatory networks of OTUD1 in immune homeostasis, inflammatory diseases, and malignant tumors. (Top) In inflammation and autoimmunity, OTUD1 exerts protective effects by deubiquitinating RIPK1 and TIPE2 (green pathways). Loss of OTUD1 expression via METTL3/YTHDF2-mediated m6A decay relieves suppression of NF-κB–NLRP3–GSDMD signaling and promotes COPD progression, whereas loss-of-function mutations (P95R, G430V) abolish IRF3 deubiquitination and drive type I interferon–mediated autoimmune diseases. (Bottom) In malignancies, OTUD1 exhibits context-dependent dual roles. As a tumor suppressor (left), it stabilizes PTEN, SMAD7, KLF4, IREB2, AIF, and DCAF10 to inhibit tumor progression, promote ferroptosis, or induce parthanatos and caspase-dependent apoptosis. Conversely, in high-grade serous ovarian cancer, pancreatic cancer, and multiple myeloma (right), OTUD1 promotes oncogenesis or alters therapeutic sensitivity via non-enzymatic aggresome–ASK1–JNK signaling or enzymatic stabilization of NRF2 and PRDX4, respectively.

In the above diseases, OTUD1 participates in inflammatory responses through regulating NF-κB or type I interferon pathways, but its functional direction shows tissue differences. In the intestine, alveoli, and systemic immune regulation, OTUD1 expression is upregulated or maintained at normal levels, inhibiting inflammatory signals through deubiquitination of RIPK1, TIPE2, or IRF3 and exerting protective effects. In the kidney and cardiac macrophages, OTUD1 expression is upregulated and activates the NF-κB pathway through stabilization of CDK9 or CARD9, promoting inflammatory factor release and exhibiting pathogenic effects. These differences may be related to the nature of inflammatory stimuli in different tissues (infectious stimuli versus mechanical/hemodynamic stress) and substrate availability (RIPK1/TIPE2/IRF3 versus CDK9/CARD9), suggesting that OTUD1 inflammatory regulatory function is not inherently protective or pathogenic but depends on the tissue microenvironment and downstream substrate repertoire (**Figure 3**).

### 2.2 Redox homeostasis and cell fate determination

#### 2.2.1 Cerebral ischemia/reperfusion injury

Cerebral ischemia/reperfusion injury is secondary damage occurring after blood flow restoration in ischemic stroke, involving oxidative stress, inflammation, and mitochondrial dysfunction; neurons are sensitive to oxidative stress damage (30–34). Allicin treatment upregulates OTUD1 expression in brain tissue (35). Mechanistically, OTUD1 removes ubiquitin chains from NRF2 through its C320 catalytic activity, prolongs its protein half-life, and promotes nuclear translocation, thereby activating downstream antioxidant response element-driven antioxidant gene transcription, reducing mitochondrial ROS levels, restoring mitochondrial membrane potential and ATP generation, and alleviating neuronal mitochondrial oxidative stress and damage. NRF2 knockdown completely eliminates the anti-mitochondrial oxidative stress and neuroprotective effects produced by OTUD1 overexpression or Allicin treatment (35, 36).

#### 2.2.2 Spinal cord injury

Spinal cord injury is severe central nervous system trauma characterized by secondary neuronal death, often leading to permanent sensory and motor dysfunction. Ferroptosis, an iron-dependent, lipid peroxidation-driven non-apoptotic form of cell death, plays a role in secondary injury following spinal cord injury (37–40). Apoptotic extracellular vesicles derived from human umbilical cord mesenchymal stem cells have neurorepair potential (41). Mechanistically, apoptotic extracellular vesicles exert anti-ferroptosis and neuroprotective effects through delivery of OTUD1; OTUD1 is highly enriched in apoptotic extracellular vesicles, and through deubiquitination it stabilizes NRF2 protein, enhances its transcriptional activity, thereby upregulating antioxidant defense systems, inhibiting neuronal ferroptosis, and promoting motor function recovery and neuroprotection (41).

#### 2.2.3 Hepatic diseases

In liver injury models, OTUD1 antioxidant and anti-cell death functions are also notable. In liver ischemia/reperfusion injury and acute hepatitis and other inflammatory liver injuries, OTUD1 mediates binding to KEAP1 and deubiquitination modification of NRF2 through its catalytic active site (C320) and ETGE motif (36). This modification process not only enhances NRF2 protein stability and nuclear translocation, but also activates downstream antioxidant response element-driven gene transcription, effectively inhibiting ROS-induced programmed cell death (oxeiptosis) and apoptosis; OTUD1-deficient mice show more severe hepatic oxidative damage, inflammation, and cell death in these models (4).

#### 2.2.4 Pancreatic ductal adenocarcinoma

Pancreatic ductal adenocarcinoma (PDAC) is a tumor type with high malignancy and poor prognosis; chemotherapy resistance is a major cause of treatment failure and high patient mortality (42). In pancreatic cancer, NRF2 is abnormally activated as a key transcription factor for antioxidant stress, and is closely associated with tumor invasiveness, angiogenesis, metastasis, and chemotherapy resistance (43). Meanwhile, YAP protein upregulation is also observed to be associated with intrinsic chemotherapy resistance in PDAC. Studies show that in PDAC cell lines with different sensitivities, the expression levels of deubiquitinases OTUD1 and DUB3 differ, and both participate in maintaining protein stability of YAP and NRF2, respectively; when OTUD1 or DUB3 is silenced, both NRF2 and YAP expression are downregulated (44). Additionally, OTUD1 performs deubiquitination modification of NRF2 through its catalytic active site (Cys320) and ETGE motif, thereby stabilizing NRF2 protein and enhancing its nuclear translocation and antioxidant function (41). In CVB3 infection models, OTUD1 overexpression can partially reverse virus-induced NRF2 expression inhibition and alleviate inflammation and ferroptosis (45). In cerebral small vessel disease-related cognitive impairment models, fluoxetine upregulates OTUD1 through Sp1-mediated transcription, activating the Nrf2/ARE pathway and exerting neuroprotective effects (46). Targeting the NRF2 pathway (such as inhibiting its activity or regulating its upstream deubiquitinase) can effectively reverse PDAC cell resistance to gemcitabine and other chemotherapy drugs, providing a potential strategy for overcoming pancreatic cancer treatment resistance (47–49).

#### 2.2.5 Colorectal cancer

Colorectal cancer (CRC) incidence and mortality rates rank among the highest globally (50, 51). Ferroptosis, an iron-dependent novel form of regulated cell death characterized by lipid peroxide accumulation, plays a role in tumorigenesis and anti-tumor immune responses (51–53). OTUD1 exerts tumor-suppressive functions in colon cancer, and its expression is selectively downregulated (54). Mechanistically, iron-responsive element-binding protein 2 (IREB2/IRP2) is a key post-transcriptional regulator of intracellular iron homeostasis, regulating mRNA stability of iron metabolism-related proteins such as transferrin receptor 1 (TFRC) by binding to iron-responsive elements (54–56). OTUD1 binds to IREB2 through its N-terminal APGR region and catalyzes removal of K48- and K63-linked ubiquitin chains from IREB2, stabilizing IREB2 protein and inducing TFRC expression upregulation, promoting cellular iron uptake (54). Increased intracellular iron content leads to elevated ROS production and triggers ferroptosis; ferroptotic tumor cells can release damage-associated molecular patterns, effectively activating host innate immune responses (54, 57). Low OTUD1 expression leads to proteasome-dependent degradation of IREB2, causing decreased TFRC expression and reduced intracellular iron content, thereby weakening ferroptosis occurrence and its accompanying anti-tumor immune response, ultimately promoting colon cancer progression (54).

#### 2.2.6 Esophageal squamous cell carcinoma

Esophageal squamous cell carcinoma (ESCC) is one of the most common digestive tract malignancies globally and is the predominant pathological type in Asia; chemotherapy resistance is a key factor causing treatment failure and poor prognosis in ESCC patients (57, 58). OTUD1 is significantly downregulated in chemotherapy-resistant ESCC cell lines compared to their parental cells, exerting chemotherapy-sensitizing effects. Mechanistically, OTUD1 enhances chemotherapy sensitivity through simultaneously regulating two different cell death pathways: on one hand, OTUD1 directly interacts with apoptosis-inducing factor (AIF), removes K63-linked ubiquitin chains at lysine 255 to promote AIF nuclear translocation and DNA binding ability, thereby activating parthanatos; simultaneously removes K27- and K63-linked ubiquitin chains at lysine 244, disrupting mitochondrial structure and impairing oxidative phosphorylation function. On the other hand, OTUD1 stabilizes DCAF10 protein to promote degradation of the anti-apoptotic protein MCL1; DCAF10, as the substrate receptor for the CUL4A-DDB1 E3 ubiquitin ligase complex, recognizes and mediates MCL1 ubiquitination, thereby triggering its proteasomal degradation and caspase-dependent classical apoptosis (57). Thus, OTUD1 exerts chemotherapy-sensitizing effects through synergistically promoting AIF-mediated non-classical parthanatos and MCL1 degradation-mediated classical apoptosis.

In the above diseases, OTUD1 regulation of redox homeostasis and cell death pathways shows duality. In cerebral ischemia/reperfusion and spinal cord injury, OTUD1 expression is upregulated or exogenously delivered, enhancing antioxidant defense through NRF2 stabilization, inhibiting neuronal ferroptosis, and exerting neuroprotective effects. In PDAC, OTUD1 similarly enhances antioxidant function through NRF2 stabilization, but this effect is converted into chemotherapy resistance and proliferation advantage in tumor cells. In CRC, low OTUD1 expression leads to IREB2 degradation, weakened ferroptosis, and enhanced tumor immune escape; in ESCC, low OTUD1 expression leads to AIF inactivation and MCL1 stabilization, increasing chemotherapy resistance. This duality indicates that OTUD1 functions through NRF2 or cell death substrates are not inherently protective or cytotoxic, but depend on the regulated cell type and tissue microenvironment (baseline oxidative stress levels and chemotherapy pressure).

### 2.3 Cell proliferation and malignant transformation

#### 2.3.1 Non-small cell lung cancer

Non-small cell lung cancer (NSCLC) accounts for approximately 85% of all lung cancer cases; although molecular targeted therapy and immunotherapy have improved prognosis in some patients, chemotherapy and targeted therapy resistance remain major causes of treatment failure (59–63). OTUD1 exerts tumor progression-inhibiting effects in NSCLC. Gene expression profiling interactive analysis(GEPIA) database analysis and clinical sample detection show that its expression is significantly lower in lung adenocarcinoma and lung squamous cell carcinoma tissues compared to normal lung tissue, and is also markedly downregulated in multiple NSCLC cell lines, with further loss in cisplatin-resistant and erlotinib-resistant cells (64). An important mechanism of its downregulation is high methylation of CpG islands in the promoter region; DNA methyltransferase inhibitor 5-Aza-dc treatment can restore OTUD1 expression (12). Mechanistically, OTUD1 inhibits NSCLC progression by stabilizing KLF4 protein (65). In cisplatin chemotherapy, OTUD1 directly binds to nucleotide excision repair system key proteins RAD23B and XPC, removes their K63-linked ubiquitin chains, and subsequently promotes E3 ubiquitin ligase PRKN-mediated K48-linked ubiquitination, thereby accelerating proteasomal degradation of the RAD23B/XPC complex, blocking DNA damage repair, and enhancing cisplatin-induced apoptosis (12). In overcoming EGFR-TKI resistance, OTUD1 functions by inhibiting YAP1 nuclear translocation and downregulating its downstream target genes SOX9 and SPP1 (64). Additionally, ATR inhibitor VE-822 has been shown to upregulate OTUD1 expression, thereby stabilizing FHL1 protein through its deubiquitinase activity to inhibit lung adenocarcinoma cell proliferation and migration (66).

#### 2.3.2 Breast cancer

Breast cancer is one of the most common malignancies in women, with highly heterogeneous biological characteristics; clinical typing is often based on molecular markers. Metastasis is the main cause of death in breast cancer patients, and TGF-β signaling is frequently reprogrammed in advanced breast cancer to promote epithelial-mesenchymal transition and tumor stem cell characteristics, thereby driving metastasis (67–71). OTUD1 serves as a key metastasis suppressor in breast cancer, identified through *in vivo* deubiquitinase library functional screening as a gene capable of inhibiting breast cancer metastasis; its low expression is closely associated with poor patient prognosis (8). Mechanistically, TGF-β signal activation induces expression of the inhibitory SMAD protein SMAD7, which serves as part of a negative feedback loop recruiting E3 ubiquitin ligase SMURF2 to the TβRI receptor to mediate receptor ubiquitination and degradation; however, SMAD7 itself is also subject to ubiquitination and degradation by E3 ligases (72). OTUD1 directly binds to SMAD7 and exerts dual deubiquitination functions: on one hand, it removes K48-linked ubiquitin chains from SMAD7 to prevent its proteasomal degradation, thereby increasing SMAD7 protein stability; on the other hand, it specifically removes K33-linked ubiquitin chains at lysine 220 of SMAD7, exposing the PY motif to enhance SMURF2 recruitment, thereby promoting TβRI degradation and ultimately effectively inhibiting TGF-β signal transduction (8). Low OTUD1 expression promotes bone and lung metastasis of breast cancer and enhances tumor cell stemness characteristics; meanwhile, OTUD1 gene copy numbers are frequently lost in multiple human cancers, and its low expression is associated with poor prognosis in breast cancer and other cancers (8).

#### 2.3.3 Clear cell renal cell carcinoma

Clear cell renal cell carcinoma (ccRCC) is the most common subtype of renal cell carcinoma; although tyrosine kinase inhibitors such as sunitinib have become standard therapy for metastatic ccRCC, most patients eventually develop acquired resistance, which is a major obstacle to improving long-term survival (73–75). OTUD1 exerts tumor progression-inhibiting effects in ccRCC. The Cancer Genome Atlas (TCGA) database analysis shows that multiple OTU family members are downregulated in tumor tissues; least absolute shrinkage and selection operator (LASSO) regression analysis identifies OTUD1 as a key gene for predicting overall survival in ccRCC patients, and its low expression is closely associated with patient prognosis deterioration. IHC tissue microarray analysis further confirms that OTUD1 protein is significantly downregulated in ccRCC tissues, with levels progressively decreasing as tumor grade increases (76). Functionally, OTUD1 overexpression significantly inhibits proliferation and induces cell cycle arrest in 786-O and ACHN cells, while overexpression of the catalytically inactive mutant lacks this effect. *In vivo* nude mouse tumorigenesis experiments show that OTUD1 knockout significantly promotes tumor growth. Mechanistically, OTUD1 directly interacts with PTEN protein, removes K48-linked ubiquitin chains from PTEN to prevent its proteasomal degradation, thereby increasing PTEN protein stability. PTEN stabilization directly leads to reduced downstream AKT phosphorylation levels and decreased expression of inflammatory factors VCAM1 and CXCL8, and the inhibitory effects of OTUD1 on cell proliferation and signaling pathways are dependent on PTEN (76). In terms of TKI resistance, OTUD1 deficiency causes PTEN degradation and subsequent persistent activation of AKT and NF-κB pathways, leading to sunitinib resistance in ccRCC cells; AKT inhibitors or NF-κB inhibitors can effectively reverse sunitinib resistance induced by OTUD1 deficiency (76).

#### 2.3.4 Ovarian cancer

High-grade serous ovarian carcinoma (HGSOC) is the most malignant and common histological subtype of ovarian cancer; although initial surgery combined with platinum-based chemotherapy is effective, approximately 80% of patients relapse and eventually develop resistance, which is closely associated with the presence of ovarian cancer stem cells (77–80). OTUD1 exerts pro-cancer functions in HGSOC, identified through integration of transcriptomics, copy number variation, and clinical survival data as a key factor for maintaining ovarian cancer stem cell characteristics. Its protein level is significantly higher in HGSOC tissues than in low-grade serous ovarian carcinoma, and high expression is associated with poor patient prognosis (14). Mechanistically, OTUD1 does not depend on its deubiquitinase activity, but mediates supramolecular self-assembly through amino acids 105–164 within the N-terminal intrinsically disordered region, forming aggregates in the cytoplasm; this process depends on microtubule system integrity. These protein aggregates serve as signaling hubs to recruit and stabilize ASK1 protein, thereby activating downstream JNK signaling pathways to maintain self-renewal and tumorigenic capacity of ovarian cancer stem cells (14). OTUD1 knockout significantly inhibits tumor sphere and soft agar colony formation abilities of ovarian cancer cells, and reduces the proportion of positive cell populations for stem cell surface markers such as CD44 and CD133. Ectopic expression of wild-type OTUD1, but not its aggregation-defective mutant, enhances cancer cell tumorigenic ability (14).

#### 2.3.5 Multiple myeloma

Multiple myeloma (MM) is a hematological malignancy characterized by clonal plasma cell malignant proliferation in the bone marrow and secretion of large amounts of abnormal monoclonal immunoglobulin (81, 82). Proteasome inhibition is an important treatment for MM, and its efficacy is closely related to the high load of immunoglobulin synthesis within plasma cells; when intracellular protein synthesis pressure is substantial, dependence on the proteasome degradation pathway increases, making cells more sensitive to proteasome inhibition (83, 84). Vdovin et al. found in MM research that OTUD1, as a deubiquitinase, regulates immunoglobulin synthesis by maintaining protein homeostasis in the endoplasmic reticulum (85). Specifically, OTUD1 deubiquitinates and stabilizes peroxiredoxin 4 (PRDX4), protecting it from endoplasmic reticulum-associated degradation pathway clearance; stabilized PRDX4 ensures normal immunoglobulin synthesis and folding in the endoplasmic reticulum. Therefore, OTUD1 expression levels are positively correlated with MM cell immunoglobulin synthesis load and M protein secretion levels. Additionally, this study confirmed the interaction between OTUD1 and KEAP1, but its function in MM remains to be clarified (85).

In NSCLC, breast cancer, and ccRCC, OTUD1 is downregulated or absent, exerting tumor progression-inhibiting effects by stabilizing tumor suppressor molecules (KLF4, SMAD7, PTEN) or inhibiting DNA repair (RAD23B/XPC); its low expression is associated with poor prognosis and resistance. In HGSOC and MM, OTUD1 is upregulated, exerting pro-cancer functions through non-enzyme-dependent aggregate formation (HGSOC) or stabilization of endoplasmic reticulum protein homeostasis factors (MM); its high expression is associated with tumor stemness maintenance and proteasome inhibitor sensitivity. This functional polarity difference is directly related to OTUD1 substrate selection in different tumors: when substrates are tumor suppressor proteins such as PTEN, SMAD7, or KLF4, the stabilizing effect of OTUD1 manifests as tumor progression inhibition; when substrates are pro-survival proteins such as ASK1 or PRDX4, it manifests as tumor progression promotion.

### 2.4 Metabolic stress and organ function remodeling

#### 2.4.1 Heart failure and myocardial infarction

While the above sections have highlighted macrophage-mediated inflammatory remodeling, hemodynamic and metabolic stress also elicit cardiomyocyte-intrinsic pathological responses. In isoproterenol, myocardial infarction, and pressure overload-induced heart failure models, OTUD1 expression is significantly upregulated in cardiomyocytes, exerting pro-pathological effects independent of macrophage infiltration (3, 11). Mechanistically, OTUD1 drives pathological remodeling through multi-substrate regulation: in cardiomyocytes, OTUD1 specifically removes K48-linked ubiquitin chains from PDE5A through its Cys320 catalytic activity, preventing its proteasomal degradation and stabilizing PDE5A protein, leading to downstream cGMP-PKG-SERCA2a signaling axis inhibition and disruption of cardiomyocyte calcium homeostasis (3); OTUD1 directly binds to PGAM5 through its OTU domain and specifically removes K63-linked ubiquitin chains from PGAM5 to stabilize PGAM5 protein, promoting downstream ASK1 phosphorylation and subsequent p38 and JNK MAPK signaling pathway activation (11); OTUD1 binds to the SH2 domain of STAT3, removes K63-linked ubiquitin chains from STAT3, promotes STAT3 Tyr705 phosphorylation and nuclear translocation, thereby upregulating hypertrophy-related gene and fibrosis factor expression (5).

#### 2.4.2 Diabetic cardiomyopathy

Diabetic cardiomyopathy is a cardiac structural and functional lesion directly induced by diabetes, independent of hypertension and coronary heart disease; myocardial energy metabolism abnormalities and mitochondrial dysfunction are its core pathological characteristics (86). OTUD1 expression is markedly elevated in diabetic cardiomyopathy lesion cardiomyocytes, mediating pathological progression of myocardial injury (87). OTUD1 binds to the AMPKα2 kinase domain and, relying on its own Cys320 catalytic site, removes K63-type ubiquitin chains at lysine residues 60 and 379 of AMPKα2; this deubiquitination modification does not affect AMPKα2 protein stability, but blocks AMPKα2 binding to upstream kinase CAMKK2, thereby inhibiting AMPK phosphorylation and enzymatic activity, causing mitochondrial complex activity decline, ROS accumulation, insufficient ATP synthesis, and ultimately inducing myocardial hypertrophy and cardiac function impairment (87).

#### 2.4.3 Myocardial ischemia/reperfusion injury

Myocardial ischemia/reperfusion injury is an important pathological process of aggravated myocardial injury following coronary recanalization, involving oxidative stress burst, intensified inflammatory response, and cardiomyocyte death (88). OTUD1 expression is upregulated in myocardial ischemia/reperfusion models, exerting pro-injury effects (89). Mechanistically, OTUD1 directly binds to RACK1 through its OTU domain and specifically removes K63-linked polyubiquitin chains from it; this deubiquitination modification significantly enhances RACK1 phosphorylation levels, thereby activating downstream MAPK and NF-κB signaling pathways, jointly promoting inflammatory factor release and cardiomyocyte apoptosis program initiation. RACK1 silencing using siRNA completely reverses in preclinical models the apoptosis-increasing effect induced by OTUD1 overexpression (89).

#### 2.4.4 Angiotensin II-induced vascular remodeling

Hypertensive vascular remodeling is an important pathological basis for hypertension-induced target organ damage, characterized by endothelial-to-mesenchymal transition, vascular wall collagen deposition, and vascular wall thickening; angiotensin II plays a central role in mediating this pathological process (90–92). OTUD1 is significantly upregulated in aortic endothelial cells of angiotensin II-infused hypertensive mouse models, exerting pro-pathological effects (9). Mechanistically, OTUD1 directly binds to the C-terminal MH2 domain of SMAD3 and simultaneously performs dual deubiquitination modification of K48- and K63-linked ubiquitin chains on SMAD3: K48 chain removal stabilizes SMAD3 protein, preventing its proteasomal degradation; K63 chain removal promotes SMAD3 and SMAD4 complex formation and subsequent nuclear translocation, both aspects jointly enhancing SMAD3 transcriptional activity to drive endothelial-to-mesenchymal transition and vascular fibrosis-related gene expression upregulation (9). SMAD3-specific inhibitors can completely block OTUD1 overexpression-induced vascular wall thickening and collagen deposition; given that OTUD1 is significantly highly expressed in endothelial cells, the OTUD1-SMAD3 signaling axis plays a central driving role in vascular endothelial dysfunction and hypertensive vascular remodeling (9).

In the cardiovascular system, OTUD1 is universally upregulated in heart failure, diabetic cardiomyopathy, myocardial ischemia/reperfusion, and hypertensive vascular remodeling, synergistically driving cardiomyocyte hypertrophy, energy metabolism disorder, apoptosis, inflammation, and vascular fibrosis through different substrates (PDE5A, PGAM5, STAT3, AMPKα2, RACK1, SMAD3). Although downstream substrates vary, OTUD1 upregulation in cardiovascular tissues is a common feature, and the common characteristic of its pro-pathological effects lies in disrupting metabolic homeostasis and promoting extracellular matrix deposition, suggesting that hemodynamic stress may be a common upstream signal inducing OTUD1 expression upregulation.

**Figure 4:**
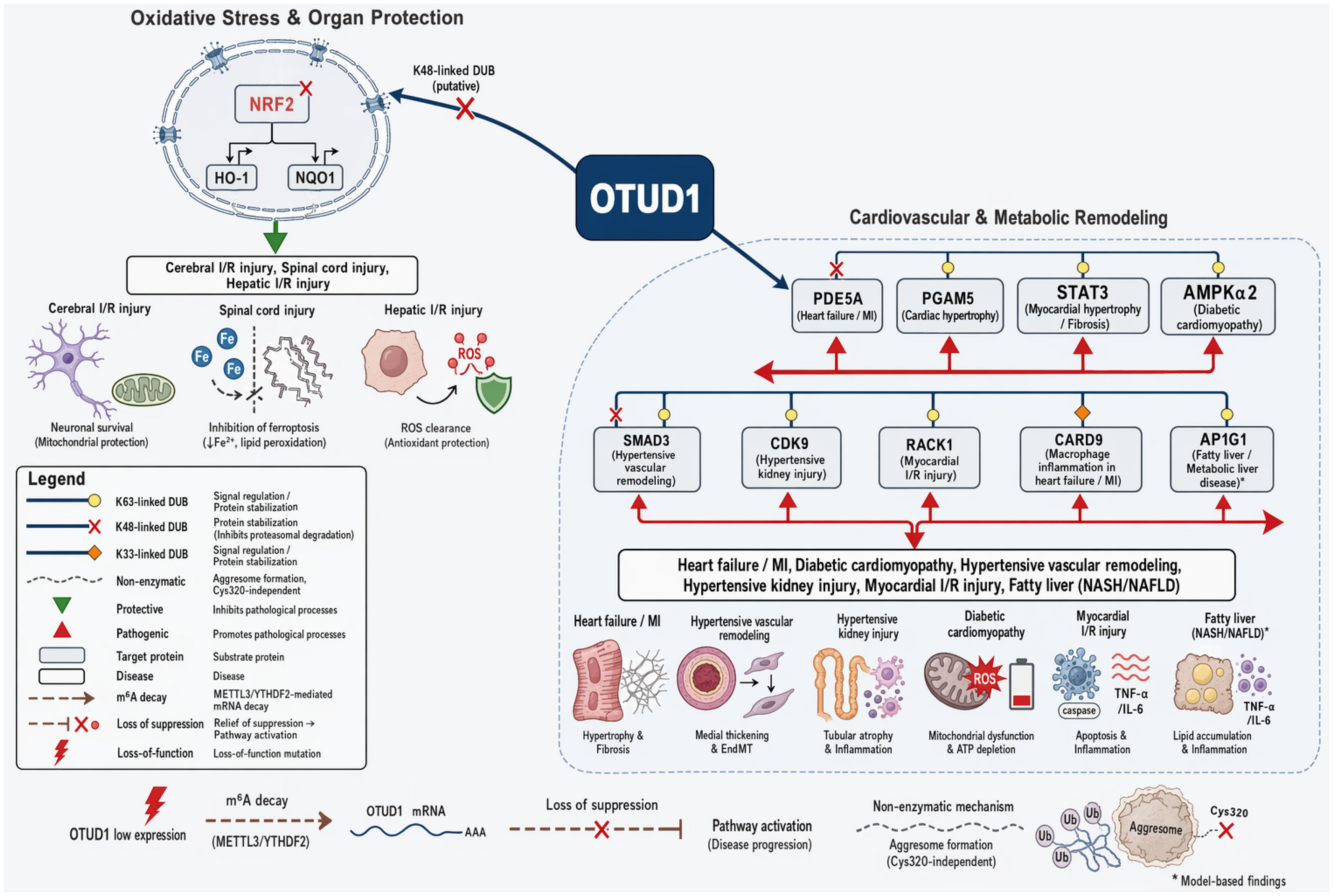
Mechanistic roles of OTUD1 in oxidative stress defense and cardiovascular-metabolic remodeling. (Left) OTUD1 directly stabilizes NRF2 through deubiquitination, promoting nuclear accumulation and antioxidant gene transcription (HO-1 and NQO1) to mitigate oxidative injury in cerebral and hepatic ischemia/reperfusion and to suppress ferroptosis in spinal cord injury. (Right) In cardiovascular and metabolic diseases, upregulated OTUD1 functions as a pathogenic driver (red pathways). Through K48-, K63-, or K33-linked deubiquitination of diverse substrates—including PDE5A, PGAM5, STAT3, AMPKα2, SMAD3, CDK9, RACK1, CARD9, and AP1G1—OTUD1 mediates cardiomyocyte apoptosis, inflammatory macrophage activation, hypertrophy, and fibrotic remodeling in heart failure, diabetic cardiomyopathy, hypertensive vascular and kidney injury, myocardial ischemia/reperfusion, and fatty liver disease.

In addition to the cardiovascular system, OTUD1 also participates in hepatic lipid metabolism stress and related inflammatory regulation. In fatty liver models, high-fat stress (palmitic acid and oleic acid) can significantly upregulate OTUD1 protein expression (93). At this time, OTUD1 interacts with AP1G1 protein and specifically removes K63-linked polyubiquitin chains from it, regulating downstream cytokine networks, thereby influencing inflammatory/immune responses and cell death processes during metabolic liver disease development (**Figure 4**).

### 2.5 Determinants of functional duality

OTUD1 exhibits dual functional characteristics of protection and pathogenicity in multiple diseases, and its functional polarity is not fixed but is primarily determined by substrate characteristics, ubiquitin chain hydrolysis preferences, and cellular and tissue microenvironment.

Substrate spectrum differences are the core mechanism of OTUD1 functional differentiation, and its biological effects are highly dependent on target protein function and cellular background. In NSCLC, breast cancer, and ccRCC, OTUD1 inhibits tumor proliferation and metastasis by stabilizing tumor suppressor proteins such as KLF4, SMAD7, and PTEN (8, 65, 76); whereas in HGSOC and MM, OTUD1 can bind and stabilize functional molecules such as ASK1 and PRDX4, activating cell pro-survival pathways, maintaining endoplasmic reticulum homeostasis, and thereby maintaining tumor stemness (14, 85). In ESCC, OTUD1 enhances tumor cell chemotherapy sensitivity by stabilizing DCAF10 to promote MCL1 degradation (58). OTUD1 shows diametrically opposite functional phenotypes in different tumors, suggesting that cell-specific adaptor molecule differences and competitive regulation by E3 ubiquitin ligases may be important reasons mediating its downstream effect differentiation.

Ubiquitin chain hydrolysis preferences further limit OTUD1 pathway selection. OTUD1 hydrolysis of K63-type ubiquitin chains primarily regulates RIPK1-NEMO, IRF3 dimerization, PGAM5-ASK1, CDK9-NF-κB, and other signaling complex assembly, participating in cell signal transduction regulation (4, 7, 10, 11, 13); whereas hydrolysis of K48-type ubiquitin chains primarily maintains stability of proteins such as PTEN, MCL1, and PDE5A, inhibiting target protein degradation (3, 58, 76). The selective hydrolysis patterns of these two ubiquitin chain types directly determine the differentiated roles of OTUD1 in signal regulation or protein stabilization in different biological pathways.

**Figure 5:**
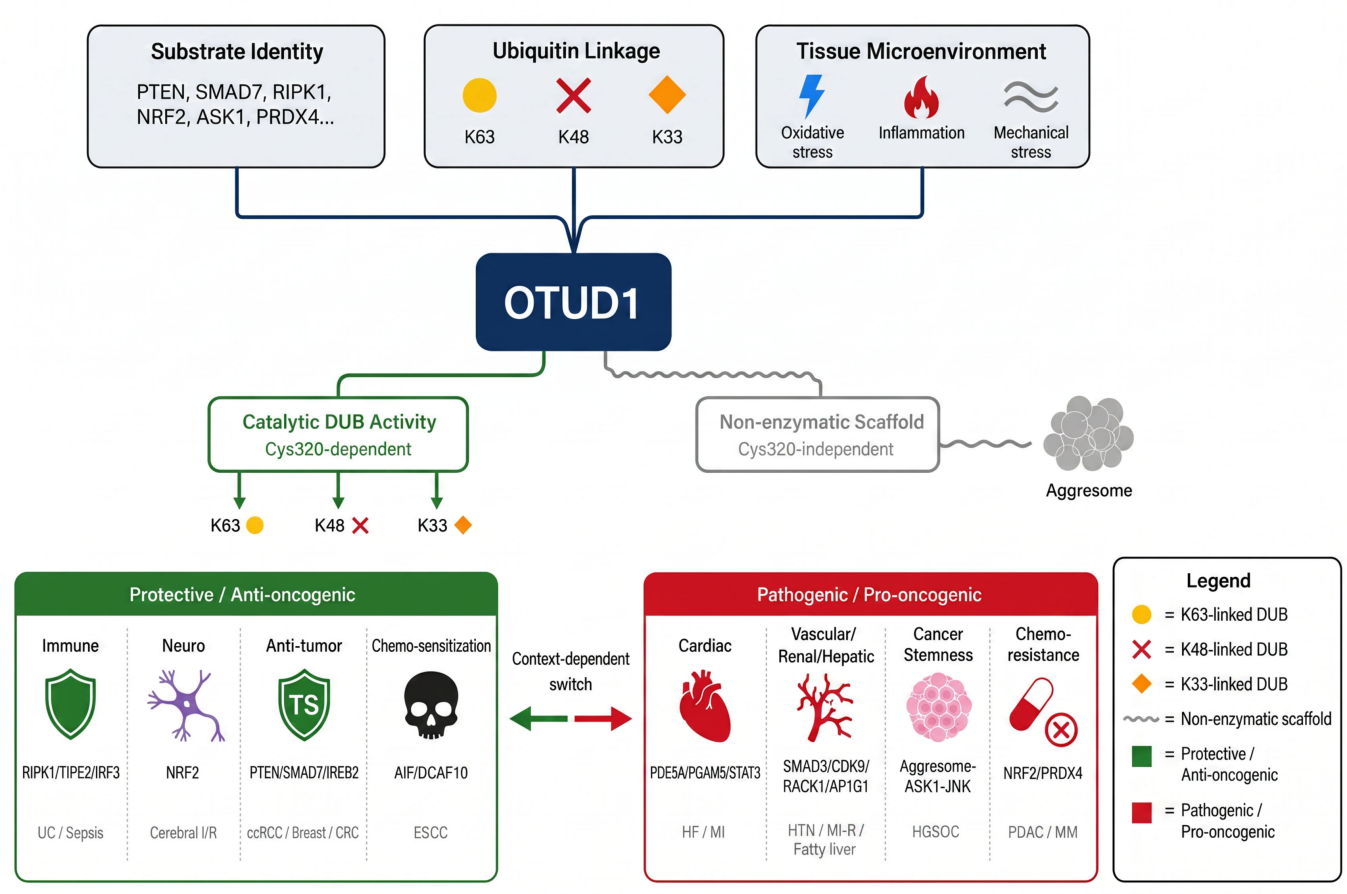
Context-dependent dual roles of OTUD1: a substrate- and linkage-dependent switch model. Three input variables—substrate identity, ubiquitin chain linkage preference (K63, K48, or K33), and tissue microenvironment (oxidative stress, inflammation, or mechanical stress)—converge on OTUD1 to determine its biological output. Cys320-dependent catalytic deubiquitination stabilizes distinct substrates and activates divergent signaling pathways, yielding either protective/anti-oncogenic (green) or pathogenic/pro-oncogenic (red) outcomes. In specific contexts, non-enzymatic N-terminal aggregation exerts scaffold functions independent of catalytic activity (aggresome–ASK1–JNK). Notably, OTUD1 expression loss via METTL3/YTHDF2-mediated m6A decay in inflammatory airway epithelium drives COPD progression, and loss-of-function mutations (P95R, G430V) abolish IRF3 deubiquitination to promote autoimmune diseases. The functional polarity is not fixed but switches according to the cellular substrate repertoire and stress milieu.

Tissue microenvironment is an important external condition regulating OTUD1 functional polarity. In neural and hepatic tissues with higher baseline oxidative stress, OTUD1 alleviates oxidative damage through NRF2 antioxidant pathway activation, exerting tissue protective effects (4, 35, 36, 41); whereas in cardiovascular tissues with high inflammatory load and mechanical stress, OTUD1 can drive inflammatory activation through the CARD9 pathway in macrophages (29), or interfere with AMPKα2-mediated metabolic regulatory networks in cardiomyocytes (87), ultimately forming multi-level pathogenic effects. Notably, HGSOC-related studies found that OTUD1 can form protein aggregates through the N-terminal intrinsically disordered region, activating the ASK1-JNK signaling pathway in a manner independent of Cys320 catalytic activity (14). This discovery of non-enzymatic function suggests that inhibitors solely targeting OTUD1 catalytic activity cannot completely block its pro-cancer effects, providing new theoretical basis for OTUD1-targeted drug development (**Figure 5**).

Collectively, OTUD1 exhibits context-dependent functional polarity across diseases. In epithelial tumors, its loss typically abrogates tumor-suppressive substrate stabilization (e.g., PTEN, SMAD7, KLF4, IREB2, AIF), whereas in high-grade serous ovarian carcinoma and multiple myeloma, its gain promotes oncogenesis via non-enzymatic scaffolding or endoplasmic reticulum proteostasis. In cardiovascular and metabolic tissues, hemodynamic and metabolic stress uniformly upregulate OTUD1, converging on hypertrophy, fibrosis, and energy dysregulation through distinct substrates (PDE5A, PGAM5, STAT3, AMPKalpha2, SMAD3, CDK9, RACK1). By contrast, in mucosal and neuronal tissues, OTUD1 predominantly exerts protective effects through NF-kappaB/IRF3 restraint or NRF2-mediated antioxidant defense. These divergent outcomes are dictated by the substrate repertoire available in each tissue, the linkage specificity of targeted ubiquitin chains (K48 for stabilization, K63 for signal regulation, K33 for dual modulation), and the nature of the prevailing stress signal. A fully unified predictive model, however, awaits systematic comparative studies across standardized cell-type and disease-stage contexts.

### 2.6 Limitations of current evidence and clinical translation bottlenecks

Current functional research on OTUD1 still has multiple controversies and inadequacies, and the limitations of existing evidence significantly constrain systematic understanding of its biological functions and clinical translation applications.

Although the dual function of OTUD1 in tumors has been widely confirmed, current studies mostly attributes functional differences simply to substrate spectrum differences. Whether confounding factors such as cell genetic background, tumor hypoxic microenvironment, and *in vitro* culture systems participate in regulating OTUD1 functional polarity still lacks direct comparative research evidence. For example, OTUD1 mediates chemotherapy resistance through NRF2/YAP pathway stabilization in pancreatic cancer (44), but whether this regulatory mode has tumor universality and whether substrate adaptor mechanisms are conserved remain to be further verified.

From the experimental system level analysis, existing cardiovascular-related studies mostly use mouse angiotensin II infusion, isoproterenol stimulation, and aortic constriction models (3, 5, 9–11, 29); animal pathological characteristics differ markedly from human cardiomyopathy and hypertension target organ damage, and the clinical reference value of experimental results is limited. Furthermore, *in vitro* experiments commonly use OTUD1 overexpression models with expression levels often significantly higher than physiological levels, which can easily cause non-specific substrate binding and abnormal functional activation, making it difficult to truly reflect endogenous OTUD1 regulatory characteristics. Bulk RNA sequencing analysis based on public databases such as TCGA cannot distinguish expression differences between tumor cells and stromal cells (64, 76), and mRNA expression levels do not completely match protein abundance and enzymatic activity, leading to insufficient evidence grades for some clinical correlation conclusions.

From the clinical translation perspective, the marked tissue functional heterogeneity of OTUD1 constitutes the core challenge for targeted intervention. Existing deubiquitinase small molecule inhibitors and ubiquitin variant drugs generally have insufficient cell penetration. More critically, although systemic OTUD1 inhibition can alleviate cardiovascular remodeling injury, it may aggravate intestinal and pulmonary mucosal inflammation (1, 13), increasing the risk of inflammatory bowel disease, COPD, and other inflammatory diseases. Therefore, avoiding off-target toxicity of systemic drug administration and developing tissue-specific gene intervention mediated by AAV, ADC/AOC precision targeted delivery, and other frontier technologies are important research directions for breaking through OTUD1 clinical translation bottlenecks and achieving safe targeted intervention (**Table 1**).

**Table 1:** Summary of expression regulation and molecular mechanisms of OTUD1 in diseases.

Disease category	Specific disease	Expression change	Core mechanism	Key experimental / clinical evidence	Experimental system	References
Tumor (tumor-suppressive)	Non-small cell lung cancer	Downregulated (promoter CpG island hypermethylation)	(1) Stabilizes KLF4 to suppress proliferation; (2) Removes K63-linked ubiquitin from RAD23B/XPC to facilitate PRKN-mediated K48 ubiquitination and proteasomal degradation, thereby impairing nucleotide excision repair and enhancing cisplatin sensitivity; (3) Inhibits YAP1 nuclear translocation to reverse EGFR-TKI resistance	Cell experiments, nude mouse tumorigenesis, clinical specimens, TCGA database; OTUD1 restoration by 5-Aza-dc	In vitro / In vivo / Human	(12)
	Breast cancer	Downregulated	Deubiquitinates SMAD7: removes K48-linked ubiquitin to prevent SMAD7 degradation and eliminates K33-linked ubiquitin to accelerate TbetaRI degradation, thus inhibiting TGF-beta-driven EMT, stemness and metastasis	In vivo functional screening, clinical samples; low expression correlates with high distant metastasis	In vivo / Human	(8)
	Clear cell renal cell carcinoma	Downregulated	Removes K48-linked ubiquitin of PTEN to stabilize PTEN and inhibit AKT/NF-kappaB cascade, improving sunitinib responsiveness	TCGA data, IHC tissue microarray, 786-O/ACHN cells, nude mouse models	In vitro / In vivo / Human	(76)
	Colorectal cancer	Downregulated	Stabilizes IREB2 to maintain intracellular iron homeostasis and promote ferroptosis; enhances anti-tumor immunity	Cell experiments, nude mouse tumorigenesis	In vitro / In vivo	(53)
	Esophageal squamous cell carcinoma	Downregulated	Deubiquitinates AIF to promote nuclear translocation and parthanatos; stabilizes DCAF10 to promote CUL4A-DDB1-mediated MCL1 ubiquitination and degradation, sensitizing cells to caspase-dependent apoptosis	Parental and cisplatin-resistant cell lines, nude mouse xenograft	In vitro / In vivo	(58)
Tumor (oncogenic-supporting)	High-grade serous ovarian carcinoma	Upregulated (aggregation-dependent)	Non-enzymatic N-terminal aggregation (amino acids 105-164) activates ASK1-JNK signaling to sustain cancer stem cell properties and cisplatin resistance	Spheroid formation, limiting dilution xenograft, clinical specimens	In vitro / In vivo / Human	(14)
	Pancreatic ductal adenocarcinoma	Upregulated	Stabilizes NRF2 and YAP to promote gemcitabine resistance and tumor progression	Chemotherapy-resistant and sensitive cell lines	In vitro	(44)
	Multiple myeloma	Upregulated	Stabilizes PRDX4 to regulate immunoglobulin synthesis and endoplasmic reticulum homeostasis; enhances proteasome inhibitor sensitivity	MM cell lines, clinical correlation	In vitro / Human	(85)
Inflammatory and	Ulcerative colitis	Loss-of-function mutation (G430V and others)	Wild-type OTUD1 removes K63-linked ubiquitin from RIPK1 to restrain NF-kappaB activation; G430V mutant loses this capacity, exacerbating inflammation	Exon sequencing of 306 patients and 300 controls; DSS colitis mouse model	In vitro / In vivo / Human	(13)
	Systemic lupus erythematosus / rheumatoid arthritis / Hashimoto thyroiditis	Loss-of-function mutation (distributed in APGR, Linker, and OTU domains)	Impaired deubiquitination of IRF3 leads to aberrant type I interferon signaling and autoimmune phenotypes	Exon sequencing of autoimmune disease cohorts	Human	(7)
	Chronic obstructive pulmonary disease	Downregulated	METTL3/YTHDF2-mediated m6A modification accelerates OTUD1 mRNA decay; reduced OTUD1 aggravates NLRP3/GSDMD-related pyroptosis in airway epithelial cells	Cigarette smoke extract-stimulated cells, public databases	In vitro / Database	(1)
	Sepsis-induced acute lung injury	Upregulated	OTUD1 stabilizes TIPE2 via K63-linked deubiquitination, restraining TAK1 phosphorylation and NF-κB/MAPK activation; reduced OTUD1 in knockout models exacerbates inflammation	Septic patients and healthy volunteers; CLP mouse model; public databases	In vitro / In vivo / Human	(19)
Neurological and neuroprotective	Cerebral ischemia/reperfusion injury	Upregulated (allicin-induced)	OTUD1-dependent NRF2 activation eliminates excessive ROS and alleviates neuronal injury via antioxidant defense	MCAO/R mouse model, HT22 OGD/R cells	In vitro / In vivo	(35)
	Spinal cord injury	Upregulated (ApoEV delivery)	NRF2-mediated ferroptosis suppression facilitates neural repair and functional recovery	Allen's weight-drop SCI model, primary neurons and microglia	In vitro / In vivo	(41)
Cardiovascular and metabolic	Heart failure and myocardial infarction	Upregulated	Stabilizes PDE5A to suppress cGMP-PKG-SERCA2a axis and disrupt calcium homeostasis; stabilizes PGAM5 to activate ASK1-JNK-p38 signaling; stabilizes STAT3 to promote hypertrophy-related gene transcription and fibrosis; macrophage CARD9 activation promotes NF-kappaB-mediated inflammatory remodeling	ISO/MI/TAC mouse models, neonatal rat cardiomyocytes, macrophages	In vitro / In vivo	(3, 5, 11)
	Hypertensive vascular remodeling	Upregulated	Dual deubiquitination of SMAD3 (K48 stabilization and K63 nuclear translocation) drives endothelial-to-mesenchymal transition and vascular fibrosis	Ang II infusion mouse model, vascular cell lines	In vitro / In vivo	(9)
	Hypertensive kidney injury	Upregulated	Deubiquitinates CDK9 at K63 to promote phosphorylation and activate NF-kappaB and inflammatory cytokine release, accelerating tubular injury and fibrosis	Ang II infusion mouse model, renal cell lines	In vitro / In vivo	(10)
	Diabetic cardiomyopathy	Upregulated	Removes K63-linked ubiquitin chains from AMPKα2 to disrupt its interaction with CAMKK2, inhibiting AMPK phosphorylation and inducing mitochondrial dysfunction and energy metabolism disorder	Diabetic mouse model, primary cardiomyocytes	In vitro / In vivo	(87)
	Myocardial ischemia/reperfusion injury	Upregulated	Removes K63-linked ubiquitin from RACK1, enhancing its phosphorylation and activating downstream MAPK/NF-κB signaling to promote inflammatory cytokine release and cardiomyocyte apoptosis	Mouse/rat I/R model, cardiomyocytes	In vitro / In vivo	(89)
Hepatic and metabolic	Hepatic ischemia/reperfusion injury	Upregulated	NRF2-mediated antioxidant defense suppresses oxeiptosis and apoptosis in hepatocytes	Hepatic I/R mouse model	In vivo	(36)
Hepatic and metabolic	Metabolic dysfunction-associated fatty liver disease	Upregulated	Removes K63-linked ubiquitin from AP1G1 to regulate lipid metabolism and hepatic steatosis	Fatty liver cell and animal models	In vitro / In vivo	(93)

## 3 Clinical translation of OTUD1: from biomarkers to precision therapeutic decision-making

### 3.1 Potential clinical applications of biomarkers

It should be noted that the current clinical significance of OTUD1 in disease diagnosis, prognostic assessment, and targeted therapy is largely derived from *in vitro* cell line experiments, animal model studies, and retrospective mining of public clinical databases. These preclinical data demonstrate certain translational potential, but their clinical efficacy and safety in the real world remain to be strictly validated through future large-scale, prospective clinical trials.

#### 3.1.1 Early diagnosis and pathological subtyping assistance

Multiple retrospective analyses based on public databases and tissue samples suggest that OTUD1 demonstrates the potential differential diagnostic value in various malignancies. In NSCLC, GEPIA database analysis and clinical sample detection show that OTUD1 mRNA and protein levels in lung adenocarcinoma and lung squamous cell carcinoma tissues are lower than those in normal lung tissue (65). In ccRCC, IHC tissue microarray analysis similarly observed low OTUD1 expression in tumor tissues (76). Conversely, in HGSOC, OTUD1 protein levels were found to be higher than those in low-grade lesions, suggesting its potential as an auxiliary differential indicator for specific subtypes (14).

In autoimmune disease susceptibility screening, OTUD1 gene mutation detection has research value. Exon sequencing by Lu et al. in 306 autoimmune disease patients identified six loss-of-function missense mutations (such as G430V) that were not detected in healthy controls (7). The G430V mutant loses its deubiquitinating capacity toward RIPK1 (13). Additionally, in COPD, dataset analysis shows that OTUD1 expression levels exhibit a progressive downward trend with increasing disease severity (GOLD stage) (1).

#### 3.1.2 Prognostic assessment and follow-up strategy optimization

In tumor prognostic assessment, preliminary analyses from multiple independent cohorts suggest that OTUD1 expression levels are correlated with patient survival outcomes. In NSCLC, low OTUD1 expression is associated with shorter overall survival (OS) and advanced TNM stage (12); OTUD1 downregulation promotes malignant cellular behaviors including enhanced migration and invasion (65). In ccRCC, LASSO regression analysis suggests that OTUD1 is one of the potential key genes for predicting patient OS, with its expression gradually decreasing as tumor grade and stage increase (76). In ovarian cancer,high OTUD1 expression is associated with shortened progression-free survival (PFS) in preclinical xenograft models and clinical cohorts, and OTUD1 expression is positively correlated with cancer stem cell marker expression (SKOV3 and OVCAR8 cells, *in vitro*, flow cytometry for CD44/CD133) (14). In breast cancer, low OTUD1 expression may indicate higher distant metastasis risk (8); whereas in MM, high OTUD1 expression is associated with immunoglobulin synthesis load and proteasome inhibitor sensitivity (85).

In non-tumor disease models, animal experiments have observed that OTUD1 expression dynamics parallel organ injury severity. In cardiovascular animal models (such as TAC surgery, isoproterenol stimulation, or myocardial infarction), myocardial tissue OTUD1 expression increases significantly with aggravated pathological remodeling (3, 11); single-cell sequencing also reveals its specific elevation in diabetic cardiomyopathy lesion cardiomyocytes (87). In angiotensin II-induced kidney injury, OTUD1 levels are positively correlated with renal tubular injury markers (Kim-1) (10). In inflammatory diseases, the low OTUD1 expression state in ulcerative colitis patient mucosa has a certain correlation with disease activity scores (13); whereas in mouse sepsis models, OTUD1 deficiency leads to sharply decreased survival rates (19).

#### 3.1.3 Experimental clues for treatment response prediction and individualized decision-making

Cell and animal model studies indicate that OTUD1 has theoretical potential as a predictive marker for chemotherapy and targeted therapy sensitivity. Regarding chemotherapy sensitivity, low OTUD1 expression is significantly associated with cisplatin resistance; cisplatin-resistant cells show approximately 3-fold higher OTUD1 promoter methylation levels (A549-CDDP versus A549, H460-CDDP versus H460, and PC9-CDDP versus PC9; *in vitro*, CCK-8 assay), whereas overexpression of OTUD1 can enhance cisplatin-induced DNA damage (γ-H2AX positive cells increase approximately 3-fold; A549 and H460 cells, *in vitro*, immunofluorescence) and apoptosis (approximately 2-fold increase in Annexin V-positive cells; A549 and H460 cells, *in vitro*, flow cytometry) (12). In ESCC, OTUD1 demonstrates chemotherapy sensitivity-enhancing effects through regulating AIF and MCL1 protein stability (58). In ovarian cancer, OTUD1-mediated stemness characteristics are associated with cisplatin resistance (14); whereas in pancreatic cancer cell lines, knockdown of deubiquitinases including OTUD1 can weaken the NRF2/YAP pathway, preliminarily showing potential for reversing gemcitabine resistance (44).

Regarding molecular targeted therapy, preclinical data provide experimental basis for individualized drug administration. In NSCLC, OTUD1 expression levels are associated with EGFR-TKI sensitivity; *in vitro* experiments show that OTUD1 overexpression restored erlotinib sensitivity in PC9-ER cells (determined by CCK-8 assay) (64); simultaneously, ATR inhibitor VE-822 was found to inhibit tumor growth through upregulating OTUD1, and the combination showed synergistic effects in mouse models (66). In ccRCC, OTUD1 deficiency-induced PTEN degradation and subsequent persistent downstream signal activation is one of the potential mechanisms causing sunitinib resistance (76). In ovarian cancer models, the small-molecule drug Ibrutinib can selectively weaken stemness of OTUD1 high-expression cells through atypical pathways (inhibiting MKK7), suggesting a marker-guided intervention strategy (14); in MM, OTUD1 expression levels are directly related to myeloma cell dependence on and sensitivity to proteasome inhibitors (85).

#### 3.1.4 Potential for dynamic monitoring of treatment response

Preclinical studies suggest that OTUD1 expression changes under specific interventions have potential for dynamic monitoring. At the epigenetic level, reversal of OTUD1 promoter methylation levels after demethylating drug (such as 5-Aza-dc) treatment in NSCLC cell lines can directly reflect the targeted effects of drugs (12). In cardiovascular pathological stress models, whether isoproterenol-stimulated cardiomyocytes or angiotensin II-stimulated endothelial cells, OTUD1 protein upregulation can occur rapidly following hemodynamic stress, suggesting its potential as an early indicator reflecting pathological stress signal activation (3, 9). Furthermore, in stem cell exosome therapy, apoptotic extracellular vesicles (ApoEVs) derived from human umbilical cord mesenchymal stem cells are highly enriched in OTUD1, and their abundance is associated with anti-ferroptosis neuroprotective effects, providing new ideas for quality control indicator development of cell therapy products (41).

### 3.2 Preliminary exploration of targeted intervention strategies

#### 3.2.1 Direct-acting tool molecules targeting OTUD1

To date, no small-molecule chemical inhibitor directly targeting the OTUD1 catalytic pocket has been approved for clinical use or entered clinical trials. However, protein-based engineered ubiquitin variants (UbVs) have been developed as preclinical probe molecules suitable for mechanistic investigation. Liu et al. employed phage display with a ubiquitin variant library to select binders against the OTUD1 OTU+UIM region (amino acids 287-481), identifying UbVOD.1 as a high-affinity binder (94). Biochemical assays demonstrated that UbVOD.1 bound to full-length OTUD1 and the OTU+UIM construct with comparable affinity (Kd approximately 1.4 nM and 0.7 nM, respectively; purified recombinant OTUD1 and OTU+UIM proteins, *in vitro*, fluorescence-based binding assay), whereas binding to the isolated OTU domain exceeded the detectable range (>20 microM; purified OTU domain alone, *in vitro*)… inhibited OTUD1 hydrolysis of Ub-AMC and K63-linked di-Ub with IC50 values of 1.2 nM and 1.1 nM, respectively (purified recombinant OTUD1, *in vitro*, fluorescence-based enzyme assay) (94). Notably, monomeric UbVOD.1 expressed in mammalian cells was unstable and failed to inhibit OTUD1 activity in global deubiquitination assays. To overcome this, tandem di-UbV fusions (UbVOD.1-OD.2 and UbVOD.2-OD.1) were constructed, which successfully inhibited OTUD1-mediated K63-linked poly-Ub chain cleavage and RIPK1 deubiquitination in HEK293 cells, without appreciable effect on K48-linked activity (94). Although these findings establish a proof-of-concept for selective OTUD1 inhibition, intracellular delivery, *in vivo* stability, and therapeutic applicability remain substantial hurdles. Future DUBTAC or PROTAC strategies may leverage high-affinity UbVOD.1 or OTUD1 amino acids 105–164 interfering peptides as recognition ligands linked to VHL or CRBN E3 ligase recruiters to achieve targeted OTUD1 degradation in pathological contexts (14, 94).

#### 3.2.2 Upstream interventions that indirectly regulate OTUD1 expression or activity

Several preclinical strategies have been identified that modulate OTUD1 abundance or activity without direct binding to the enzyme. (i) Epigenetic modulators: the DNA methyltransferase inhibitor 5-Aza-dc restored OTUD1 expression in NSCLC cell lines (A549, H460, PC9, and others) by reversing promoter CpG island hypermethylation (12). (ii) Kinase inhibitors: VE-822, an ATR inhibitor identified via CellMiner database screening, upregulated OTUD1 protein levels in HCC827, H460, H1965, and H2030 lung adenocarcinoma cells, thereby stabilizing FHL1 and inhibiting tumor growth (66). (iii) Natural compounds: allicin upregulated OTUD1 in cerebral ischemia/reperfusion models (35); in ccRCC cell models, melatonin induced OTUD1 expression via Sp1-mediated transcriptional activation (95). (iv) Post-transcriptional modulation: knockdown of METTL3 or YTHDF2 restored OTUD1 expression in COPD airway epithelial cells by blocking m6A-dependent mRNA decay (1). These interventions offer indirect routes to restore OTUD1 in deficiency states, though their specificity for OTUD1 relative to other cellular targets is limited.

#### 3.2.3 Pathway-level interventions targeting OTUD1-related signaling

Given the absence of clinically viable direct OTUD1 inhibitors, a pragmatic translational approach involves targeting downstream effectors or compensatory pathways. In high-grade serous ovarian carcinoma, ibrutinib (a MKK7-JNK pathway inhibitor) attenuated cancer stem cell properties sustained by OTUD1-ASK1 aggregation (14); selonsertib, SP600125, and IN-8 (JNK inhibitors) similarly reduced spheroid formation and tumorigenicity in preclinical models (14). In cardiovascular disease, GS-444217 (an ASK1 inhibitor) reversed myocardial hypertrophy driven by OTUD1-PGAM5 signaling (11); sildenafil (PDE5A modulator) improved cardiac dysfunction associated with OTUD1 upregulation (3); NVP-2 (CDK9 inhibitor) alleviated renal tubular injury and fibrosis (10); and SIS3 (SMAD3 inhibitor) blocked vascular endothelial-to-mesenchymal transition and wall thickening (9). These agents do not bind OTUD1 directly but modulate downstream pathological cascades, offering a functionally indirect yet clinically more accessible translational strategy.

### 3.3 Core bottlenecks and future directions for clinical translation

Across existing literature, the clinical translation of OTUD1 still faces notable technical challenges: first, highly selective small-molecule direct probes with high cell penetrability are extremely scarce; second, the vast majority of evidence is limited to animal experiments or single-center cell bank analyses, lacking validation by multi-center, large-sample prospective clinical cohorts; third, the discovery of non-enzymatic scaffold functions challenges traditional small-molecule drug development paradigms (14).

To break through these bottlenecks, future translational medicine research should focus on the following directions: first, advancing organ/tissue-specific delivery technologies. To avoid systemic intervention toxicity, active development of gene therapy based on AAV vectors (such as cardiomyocyte-specific AAV9) (11), local drug delivery systems targeting the digestive tract mucosa, or antibody-drug conjugates/nucleic acid-drug conjugates (ADC/AOC) should be pursued. Second, establishing rigorous molecular stratification and monitoring systems. In any future intervention trials involving OTUD1 upstream or downstream pathways, in addition to evaluating primary efficacy indicators, intestinal microecological inflammation, cardiovascular functional status, and antiviral immune function must be included as mandatory systemic safety monitoring parameters(**Table 2**).

**Table 2:** Clinical translational potential of OTUD1.

Target disease	Biomarker / therapeutic strategy	Molecular mechanism	Clinical implication	Preclinical evidence level	References
Non-small cell lung cancer	Prognostic biomarker: low OTUD1 expression correlates with advanced stage and poor prognosis	Promoter CpG island hypermethylation silences OTUD1; low OTUD1 stabilizes RAD23B/XPC complex, enhancing nucleotide excision repair	Potential biomarker for tumor staging and prognostic stratification; suggests potential for predicting cisplatin resistance in preclinical settings	TCGA pan-cancer database mining; qRT-PCR and western blot of 12 paired clinical specimens; IHC tissue microarray (TJMUCH cohort); Kaplan-Meier analysis (GEO GSE41271); RNA-seq of H460 parental versus cisplatin-resistant cells	(12)
Non-small cell lung cancer	5-Aza-dc restores OTUD1 expression	DNMT inhibition reverses promoter hypermethylation, rescuing OTUD1 transcription	Preclinical evidence for epigenetic priming to resensitize cisplatin in preclinical models	A549, H460, PC9, H292, H520, H1703, SPC-A1, H1299, Calu-6, H1965, H661, H358, H226, and Calu-3 cells *in vitro* (5 microM for 95 h or 10 microM for 72 h, qRT-PCR)	(12)
Non-small cell lung cancer	OTUD1 overexpression enhances cisplatin sensitivity	Cleaves K63-linked ubiquitin from RAD23B and XPC; promotes PRKN-mediated K48 ubiquitination and proteasomal degradation of RAD23B/XPC	Impairs DNA damage repair; potentiates cisplatin-induced apoptosis in cell and animal models	A549 and H460 cells *in vitro* (CCK-8, Annexin V/PI, comet assay, western blot); female BALB/c nude mice xenograft (1 times 10^6 H460 cells, inguinal injection, CDDP 5 mg/kg i.p. on days 14, 18, 22, 26; 30-day observation)	(12)
Lung adenocarcinoma	VE-822 upregulates OTUD1 to stabilize FHL1	ATR inhibitor transcriptionally upregulates OTUD1, which deubiquitinates and stabilizes FHL1	Inhibits proliferation, migration, and invasion in preclinical models	HCC827 and NCI-H2030 cells in vitro (CCK-8, wound healing, Transwell); BALB/c nude mice xenograft (5 times 10^6 H827 cells, subcutaneous bilateral flanks, 5 weeks); C57BL/6J WT versus Otud1-KO primary urethane-induced lung cancer model (800 mg/kg i.p., twice weekly for 5 weeks); combined VE-822–60 mg/kg oral gavage daily for 21 days starting day 14	(66)
Breast cancer	Low OTUD1 predicts high metastatic risk and unfavorable prognosis	OTUD1 depletion leads to aberrant TGF-beta activation and EMT progression	Preoperative metastatic risk evaluation and adjuvant regimen optimization (preclinical evidence)	MDA-MB-231, MCF10A-RAS, MCF7, 4T1, and 4T07 cells *in vitro* (shRNA screening, Transwell, 3D Matrigel); BALB/c nude mice *in vivo* (cardiac injection of MDA-MB-231-Luc/GFP 1 times 10^5 for bone metastasis; tail vein injection of 4T1-Luc or 4T07-Luc 1 times 10^5 for lung metastasis; mammary fat pad injection of 4T1-Luc 1 times 10^5 for orthotopic growth); NKI295 and TCGA database analysis	(8)
Clear cell renal cell carcinoma	Low OTUD1 indicates sunitinib resistance; higher OTUD1 correlates with better TKI responsiveness	Downregulated OTUD1 induces PTEN degradation and persistent AKT/NF-kappaB activation	Potential drug susceptibility indicator; AKT/NF-kappaB inhibitors reverse acquired sunitinib resistance in cell and animal models	786-O and ACHN cells *in vitro* (CCK-8, flow cytometry, western blot); BALB/c nude mice xenograft (1 times 10^7 786-O cells, subcutaneous, 5 mice per group); TCGA-KIRC database; IHC tissue microarray (38 tumors and 38 normal tissues)	(76)
Colorectal cancer	Low OTUD1 predicts ferroptosis escape and compromised anti-tumor immunity	Reduced IREB2 stability decreases intracellular iron uptake	Prognostic assessment; preclinical data suggest upregulating OTUD1 may synergize with chemotherapy via inducing ferroptosis	HCT116, SW480, and DLD1 cells *in vitro* (CCK-8, iron content, ROS, lipid peroxidation, flow cytometry); BALB/c nude mice xenograft (HCT116 cells, subcutaneous)	(53)
Esophageal squamous cell carcinoma	Reduced OTUD1 predicts chemotherapy resistance	Impaired AIF nuclear translocation plus accumulated anti-apoptotic MCL1	Chemotherapy sensitivity screening in preclinical models	KYSE150, KYSE450, and other ESCC parental versus cisplatin-resistant cells *in vitro* (CCK-8, flow cytometry for apoptosis, immunofluorescence for AIF nuclear translocation, mitochondrial function); BALB/c nude mice xenograft (subcutaneous)	(58)
High-grade serous ovarian carcinoma	High OTUD1 denotes robust stemness and cisplatin resistance; ibrutinib indirectly blocks downstream JNK to counter oncogenic effect	OTUD1 aggregation activates ASK1-JNK to sustain cancer stem cell properties; ibrutinib inhibits MKK7-JNK	Prognostic stratification; combined chemotherapy regimen design supported by preclinical models	SKOV3, OVCAR8, CAOV3, and OVCAR3 cells *in vitro* (spheroid formation assay, soft agar colony formation, flow cytometry for CD44/CD133); BALB/c nude mice xenograft (SKOV3 sgCtrl/sgOTUD1–5 times 10^6; OVCAR3 sgCtrl/sgOTUD1–1 times 10^7; OVCAR8–1 times 10^7; limiting dilution assay SKOV3 EV/OTUD1-WT/OTUD1-Delta105-164–1 times 10^4 to 1 times 10^6); ibrutinib 25 mg/kg every other day i.p.	(14)
Pancreatic ductal adenocarcinoma	High NRF2 level implies gemcitabine resistance; OTUD1 suppression downregulates NRF2/YAP	OTUD1-dependent NRF2/YAP stabilization mediates chemoresistance	Predict gemcitabine efficacy in preclinical models; supports development of DUB-targeted inhibitors	PANC-1 and other chemotherapy-resistant versus sensitive PDAC cells *in vitro* (western blot, cell proliferation, siRNA knockdown of OTUD1 and USP17); no animal model reported	(44)
Multiple myeloma	Elevated OTUD1 is associated with better response to proteasome inhibitors	Regulates PRDX4 stability, immunoglobulin synthesis, and ER homeostasis	Potential for individualized proteasome inhibitor administration and related proteasome inhibitors (preclinical evidence)	MM.1S and RPMI8226 cells *in vitro* (western blot, immunoglobulin detection, proliferation, proteasome inhibitor sensitivity); no animal model reported	(85)
Ulcerative colitis	Loss-of-function OTUD1 mutation increases disease susceptibility; OTUD1 upregulation suppresses NF-kappaB-mediated inflammation	G430V mutant loses RIPK1-targeted deubiquitinating capacity	Genetic screening for high-risk population; intestinal-specific OTUD1 upregulation for disease intervention (preclinical)	Exon sequencing of 306 autoimmune disease patients (87 SLE, 110 RA, 87 UC, 22 HT) versus 300 healthy controls (China-Japan Friendship Hospital); U937, THP-1, and BMDMs *in vitro* (LPS stimulation, ELISA, flow cytometry); C57BL/6 mice DSS colitis model (2.5% DSS, 6 days); UC microarray datasets (GEO, EBI Array Express)	(13)
Chronic obstructive pulmonary disease	Progressive OTUD1 reduction parallels increased GOLD grading	m6A-dependent OTUD1 degradation aggravates NLRP3/GSDMD-related pyroptosis	Dynamic monitoring of disease severity during follow-up (preclinical)	BEAS-2B cells *in vitro* (10% cigarette smoke extract, 0–48 h); public databases GSE38964 and GSE69818; no animal model reported	(1)
Sepsis-induced acute lung injury	OTUD1 upregulation in peripheral blood exerts immunoregulatory effects; OTUD1 deficiency in preclinical models predicts excessive inflammatory response and sharply decreased survival rates	Reduced TIPE2 stability causes unrestrained NF-kappaB overactivation	Potential early warning indicator for severe sepsis (preclinical) and prognostic judgement (preclinical)	20 septic patients and 20 healthy volunteers (Renmin Hospital of Wuhan University); C57BL/6 mice CLP model (n=10-15); BMDMs *in vitro* (LPS 1 microg/mL); public databases GSE28750, GSE76293, GSE57065	(19)
Cerebral ischemia/reperfusion injury	Allicin upregulates OTUD1/NRF2 pathway	OTUD1-dependent NRF2 activation eliminates excessive ROS and alleviates neuronal injury	Preclinical development of neuroprotective small-molecule drugs	C57BL/6J mice MCAO/R model (60 min occlusion, 2 weeks); HT22 cells *in vitro* (OGD/R, 2 h hypoxia/12 h reoxygenation); allicin 50 mg/kg/d i.p.; AAV-hSyn-OTUD1 stereotactic cortical injection	(35)
Spinal cord injury	ApoEV-based delivery to enrich intracellular OTUD1	NRF2-mediated ferroptosis suppression facilitates neural repair	Quality control marker for MSC-derived vesicle therapeutics (preclinical)	C57BL/6J mice Allen's weight-drop SCI model (T10, 30 g/cm); primary cortical neurons and microglia from P1 C57BL/6J mice; UC-MSC ApoEVs (50 or 100 microg/mouse, tail vein); AAV-Syn-shNRF2 stereotactic injection	(41)
Heart failure and myocardial infarction	PDE5A modulator (sildenafil) for isoproterenol- and infarction-induced dysfunction; ASK1 inhibitor (GS-444217) for pressure-overload hypertrophy; STAT3 inhibitor (stattic) for hemodynamic-stress-induced inflammatory remodeling	Stabilizes PDE5A to suppress cGMP-PKG-SERCA2a axis and disrupt calcium homeostasis; stabilizes PGAM5 to activate ASK1-JNK-p38 signaling; stabilizes STAT3 to promote hypertrophy-related gene transcription and fibrosis; macrophage activation promotes NF-kappaB-mediated inflammatory remodeling	Attenuates cardiac hypertrophy and fibrosis in preclinical models; tissue-specific AAV9 delivery may avoid systemic adverse effects	8-week-old male C57BL/6 mice, isoproterenol 30 mg/kg/d via Alzet osmotic pump for 2 weeks or LAD ligation myocardial infarction; neonatal rat ventricular myocytes and H9c2 cells *in vitro*. 6-8-week-old male C57BL/6 mice, Ang II 1 microg/kg/min via Alzet osmotic pump for 4 weeks or transverse aortic constriction for 4 weeks; neonatal rat ventricular myocytes, H9c2, and mouse peritoneal macrophages *in vitro*. Otud1-CKO mice with transverse aortic constriction for 4 weeks; AAV9-Otud1 tail vein injection; neonatal rat cardiomyocytes and AC16 cells *in vitro*.	(3, 5, 11)
Hypertensive vascular remodeling	SMAD3 inhibitor (SIS3)	Dual deubiquitination of SMAD3 (K48 stabilization and K63 nuclear translocation) drives endothelial-to-mesenchymal transition and vascular fibrosis	Reverses vascular wall thickening and collagen deposition in preclinical models	8-week-old male C57BL/6 mice, Ang II 1000 ng/kg/min via osmotic pump for 4 weeks; HUVECs, MOVAS, and bEnd.3 cells *in vitro*.	(9)
Hypertensive kidney injury	CDK9 inhibitor (NVP-2)	Deubiquitinates CDK9 at K63 to promote phosphorylation and activate NF-kappaB and inflammatory cytokine release	Alleviates renal tubular injury and fibrosis in preclinical models	8-week-old male C57BL/6 mice, Ang II 1 microg/kg/min for 4 weeks; AAV9-OTUD1 tail vein injection (2 times 10^11 vg/mouse/month, 2 times); TCMK-1 and SV40 MES-13 cells *in vitro*.	(10)

## 4 Summary and outlook

OTUD1 is a deubiquitinase containing a conserved OTU catalytic domain, with K63-linked ubiquitin chains as its primary substrate. It participates in multiple core biological processes including immune inflammation, oxidative stress, cell death, and energy metabolism through regulating substrate protein ubiquitination status. Its physiological functions are concentrated in maintaining immune homeostasis of NF-κB and type I interferon pathways, KEAP1-NRF2 antioxidant balance, and balanced regulation of cell survival and death. Under disease conditions, OTUD1 functional polarity is highly dependent on tissue microenvironment: in the nervous system, it primarily exerts neuroprotective effects through NRF2 stabilization; in various epithelial tumors, it is frequently downregulated due to promoter methylation or transcriptional inhibition, restricting malignant progression through tumor suppressor protein stabilization; whereas in tumor types such as HGSOC, PDAC, and MM, its expression is upregulated, maintaining tumor stemness and survival advantages through non-enzymatic aggregate formation or endoplasmic reticulum protein stabilization; in cardiovascular and metabolic diseases, OTUD1 is generally upregulated in response to hemodynamic or metabolic stress, promoting myocardial hypertrophy, fibrosis, and vascular remodeling through stabilization of substrates such as PDE5A, PGAM5, STAT3, AMPKα2, or SMAD3; in intestinal and pulmonary inflammation, it inhibits excessive inflammation through deubiquitination of RIPK1, TIPE2, or IRF3, exerting protective regulation.

Despite substantial preclinical progress, several challenges constrain the translational trajectory of OTUD1-targeted interventions. First, the absence of small-molecule inhibitors with suitable pharmacokinetic profiles necessitates continued development of engineered ubiquitin variants or PROTAC-based degradation strategies, though intracellular delivery and tissue specificity remain substantial hurdles. Second, the functional duality of OTUD1—tumor-suppressive in some malignancies yet oncogenic-supporting in others—demands rigorous patient stratification based on disease subtype, tissue context, and substrate availability prior to any therapeutic application. Third, most current evidence derives from cell lines and rodent models; prospective clinical validation of OTUD1 as a prognostic biomarker or drug response predictor requires large-cohort retrospective analyses and, ultimately, randomized controlled trials. Finally, the non-enzymatic scaffolding functions of OTUD1, particularly in high-grade serous ovarian carcinoma, cannot be addressed by catalytic inhibitors alone, underscoring the need for orthogonal therapeutic modalities. Resolving these issues will be essential to translate OTUD1 biology from mechanistic understanding to clinical benefit.

Future research should prioritize the following directions: at the basic mechanism level, using proximity labeling, mass spectrometry proteomics, and organoid models to systematically analyze OTUD1 tissue-specific interaction networks, and clarifying dynamic changes in its substrate repertoire under different disease states; at the clinical translation level, establishing standardized OTUD1 protein detection and activity assessment systems, and conducting retrospective efficacy analyses and prospective biomarker validation for tumors and cardiovascular diseases; at the drug development level, developing dual-function inhibitors targeting both OTUD1 catalytic activity and protein-protein interaction interfaces, and exploring AAV-mediated tissue-specific gene intervention, PROTAC degradation technology, and inhalation or intestinal local drug delivery systems to avoid off-target risks of systemic intervention. Advancement of these studies will help improve understanding of OTUD1 regulatory networks, and provide support for its translation to clinical diagnosis and precision therapy.

## Glossary

5-Aza-dc: 5-aza-2'-deoxycytidine
AAV: adeno-associated virus
ADC: antibody-drug conjugate
AIF: apoptosis-inducing factor
AKT: protein kinase B
AMPKα2: AMP-activated protein kinase alpha 2
Ang II: angiotensin II
AOC: antibody-oligonucleotide conjugate
AP1G1: adaptor related protein complex 1 subunit gamma 1
APGR: Ala-, Pro-, and Gly-rich region
ApoEVs: apoptotic extracellular vesicles
ARE: antioxidant response element
ASK1: apoptosis signal-regulating kinase 1
ATR: ataxia telangiectasia and Rad3-related
BCL10: B-cell lymphoma/leukemia 10
CAMKK2: calcium/calmodulin-dependent protein kinase kinase 2
CARD9: caspase recruitment domain family member 9
ccRCC: clear cell renal cell carcinoma
CD44: cluster of differentiation 44
CDK9: cyclin-dependent kinase 9
CLP: cecal ligation and puncture
COPD: chronic obstructive pulmonary disease
CRC: colorectal cancer
CSC: cancer stem cell
CUL3: Cullin 3
CUL4A: Cullin 4A
CVB3: Coxsackievirus B3
Cys320: cysteine 320
DCAF10: DDB1 and CUL4 associated factor 10
DDB1: DNA damage-binding protein 1
DSS: dextran sulfate sodium
DUB: deubiquitinase
EGFR-TKI: epidermal growth factor receptor tyrosine kinase inhibitor
EMT: epithelial-mesenchymal transition
EndMT: endothelial-to-mesenchymal transition
ER: endoplasmic reticulum
ESCC: esophageal squamous cell carcinoma
ETGE: glutamate-threonine-glycine-glutamate motif
FADD: Fas-associated death domain
FHL1: four and a half LIM domains 1
FOXO3: forkhead box O3
G430V: glycine 430 to valine
GEPIA: gene expression profiling interactive analysis
GOLD: global initiative for chronic obstructive lung disease
GSDMD: gasdermin D
HGSOC: high-grade serous ovarian carcinoma
HO-1: heme oxygenase-1
IFN-β: interferon beta
Ig: immunoglobulin
IHC: immunohistochemistry
IKKγ: inhibitor of nuclear factor kappa-B kinase subunit gamma
IL-1β: interleukin-1 beta
IL-6: interleukin-6
I/R: ischemia/reperfusion
IRAK1: interleukin-1 receptor-associated kinase 1
IREB2: iron-responsive element-binding protein 2
IRF3: interferon regulatory factor 3
JNK: c-Jun N-terminal kinase
KEAP1: Kelch-like ECH-associated protein 1
Kim-1: kidney injury molecule-1
KLF4: Krüppel-like factor 4
LPS: lipopolysaccharide
LUBAC: linear ubiquitin chain assembly complex
MALT1: mucosa-associated lymphoid tissue lymphoma translocation protein 1
MAPK: mitogen-activated protein kinase
m6A: N6-methyladenosine
METTL3: methyltransferase-like 3
MI: myocardial infarction
MLKL: mixed lineage kinase domain-like
MM: multiple myeloma
NAFLD: non-alcoholic fatty liver disease
NASH: non-alcoholic steatohepatitis
NEMO: NF-κB essential modulator
NF-κB: nuclear factor kappa B
NLRP3: NOD-, LRR- and pyrin domain-containing protein 3
NQO1: NAD(P)H quinone dehydrogenase 1
NSCLC: non-small cell lung cancer
NRF2: nuclear factor erythroid 2-related factor 2
OUT: ovarian tumor
OTUD1: ovarian tumor domain-containing protein 1
P95R: proline 95 to arginine
PDAC: pancreatic ductal adenocarcinoma
PDE5A: phosphodiesterase 5A
PGAM5: phosphoglycerate mutase family member 5
poly(I:C): polyinosinic:polycytidylic acid
PRDX4: peroxiredoxin 4
PRKN: parkin
PROTAC: proteolysis-targeting chimera
PTEN: phosphatase and tensin homolog
RACK1: receptor for activated C kinase 1
RAD23B: RAD23 homolog B
RIPK1: receptor-interacting protein kinase 1
RIPK3: receptor-interacting protein kinase 3
ROS: reactive oxygen species
SCI: spinal cord injury
SeV: Sendai virus
SH2: Src homology 2
SIS3: specific inhibitor of Smad3
SMAD3: SMAD family member 3
SMAD4: SMAD family member 4
SMAD7: SMAD family member 7
SMURF2: SMAD ubiquitination regulatory factor 2
SOX9: SRY-box transcription factor 9
SPP1: secreted phosphoprotein 1
STAT3: signal transducer and activator of transcription 3
TAC: transverse aortic constriction
TAK1: transforming growth factor beta-activated kinase 1
TCGA: the cancer genome atlas
TGF-β: transforming growth factor beta
TβRI: TGF-β receptor type I
TIPE2: tumor necrosis factor alpha-induced protein 8-like 2
TKI: tyrosine kinase inhibitor
TNF-α: tumor necrosis factor alpha
UIM: ubiquitin-interacting motif
UC: ulcerative colitis
UC-MSCs: umbilical cord mesenchymal stem cells
VCAM1: vascular cell adhesion molecule 1
XPC: xeroderma pigmentosum group C
YAP1: yes-associated protein 1
YTHDF1/2: YTH domain family 1/2

## References

[1] Gao J Shen Z Tian W Xia J Cao W Chen Z . METTL3-mediated m6A methylation and its impact on OTUD1 expression in chronic obstructive pulmonary disease. Mol Med Rep. (2025) 32:206. doi: 10.3892/mmr.2025.13571 40417884 PMC12117359

[2] Oikawa D Shimizu K Tokunaga F . Pleiotropic roles of a KEAP1-associated deubiquitinase, OTUD1. Antioxidants (Basel). (2023) 12:350. doi: 10.3390/antiox12020350 36829909 PMC9952104

[3] Wang Q Liang S Qian J Xu J Zheng Q Wang M . OTUD1 promotes isoprenaline- and myocardial infarction-induced heart failure by targeting PDE5A in cardiomyocytes. Biochim Biophys Acta Mol Basis Dis. (2024) 1870:167018. doi: 10.1016/j.bbadis.2024.167018 38185350

[4] Oikawa D Gi M Kosako H Shimizu K Takahashi H Shiota M . OTUD1 deubiquitinase regulates NF-κB- and KEAP1-mediated inflammatory responses and reactive oxygen species-associated cell death pathways. Cell Death Dis. (2022) 13:694. doi: 10.1038/s41419-022-05145-5 35941131 PMC9360000

[5] Wang M Han X Yu T Wang M Luo W Zou C . OTUD1 promotes pathological cardiac remodeling and heart failure by targeting STAT3 in cardiomyocytes. Theranostics. (2023) 13:2263–80. doi: 10.7150/thno.83340 37153745 PMC10157730

[6] Mevissen TE Hospenthal MK Geurink PP Elliott PR Akutsu M Arnaudo N . OTU deubiquitinases reveal mechanisms of linkage specificity and enable ubiquitin chain restriction analysis. Cell. (2013) 154:169–84. doi: 10.1016/j.cell.2013.05.046 23827681 PMC3705208

[7] Lu D Song J Sun Y Qi F Liu L Jin Y . Mutations of deubiquitinase OTUD1 are associated with autoimmune disorders. J Autoimmun. (2018) 94:156–65. doi: 10.1016/j.jaut.2018.07.019 30100102

[8] Zhang Z Fan Y Xie F Zhou H Jin K Shao L . Breast cancer metastasis suppressor OTUD1 deubiquitinates SMAD7. Nat Commun. (2017) 8:2116. doi: 10.1038/s41467-017-02029-7 29235476 PMC5727433

[9] Huang Z Shen S Wang M Li W Wu G Huang W . Mouse endothelial OTUD1 promotes angiotensin II-induced vascular remodeling by deubiquitinating SMAD3. EMBO Rep. (2023) 24:e56135. doi: 10.15252/embr.202256135 36579465 PMC9986815

[10] Wang MY Yu TX Wang QY Han X Hu X Ye SJ . OTUD1 promotes hypertensive kidney fibrosis and injury by deubiquitinating CDK9 in renal epithelial cells. Acta Pharmacol Sin. (2024) 45:765–76. doi: 10.1038/s41401-023-01192-6 38110583 PMC10943066

[11] Huang K Sun X Xu X Lu J Zhang B Li Q . METTL3-mediated m6A modification of OTUD1 aggravates press overload induced myocardial hypertrophy by deubiquitinating PGAM5. Int J Biol Sci. (2024) 20:4908–21. doi: 10.7150/ijbs.95707 39309432 PMC11414395

[12] Wu Z Wang M Dong X Sun Y Zhang L Tang M . The deubiquitinase OTUD1 orchestrates cisplatin chemosensitivity of non-small cell lung cancer through destabilizing RAD23B/XPC. Oncogene. (2025) 44:4855–67. doi: 10.1038/s41388-025-03647-y 41286308

[13] Wu B Qiang L Zhang Y Fu Y Zhao M Lei Z . The deubiquitinase OTUD1 inhibits colonic inflammation by suppressing RIPK1-mediated NF-κB signaling. Cell Mol Immunol. (2022) 19:276–89. doi: 10.1038/s41423-021-00810-9 34876703 PMC8803853

[14] Chen Y Qiang Y Fan J Zheng Q Yan L Fan G . Aggresome formation promotes ASK1/JNK signaling activation and stemness maintenance in ovarian cancer. Nat Commun. (2024) 15:1321. doi: 10.1038/s41467-024-45698-x 38351029 PMC10864366

[15] Zhu YL Liu CZ Li Y . Regulatory role of protein ubiquitination in the pathogenesis and progression of ulcerative colitis. J Dig Dis. (2025) 26:398–405. doi: 10.1111/1751-2980.70017 41236013

[16] Chen H Hu Q Wen T Luo L Liu L Wang L . Arteannuin B, a sesquiterpene lactone from Artemisia annua, attenuates inflammatory response by inhibiting the ubiquitin-conjugating enzyme UBE2D3-mediated NF-κB activation. Phytomedicine. (2024) 124:155263. doi: 10.1016/j.phymed.2023.155263 38181532

[17] Matthay MA Zemans RL Zimmerman GA Arabi YM Beitler JR Mercat A . Acute respiratory distress syndrome. Nat Rev Dis Primers. (2019) 5:18. doi: 10.1038/s41572-019-0069-0 30872586 PMC6709677

[18] Gotts JE Matthay MA . Sepsis: pathophysiology and clinical management. BMJ. (2016) 353:i1585. doi: 10.1136/bmj.i1585 27217054

[19] Ming T Liu H Yuan M Tian J Fang Q Liu Y . The deubiquitinase OTUD1 deubiquitinates TIPE2 and plays a protective role in sepsis-induced lung injury by targeting TAK1-mediated MAPK and NF-κB signaling. Biochem Pharmacol. (2024) 227:116418. doi: 10.1016/j.bcp.2024.116418 38996928

[20] Agustí A Celli BR Criner GJ Halpin D Anzueto A Barnes P . Global initiative for chronic obstructive lung disease 2023 report: GOLD executive summary. Eur Respir J. (2023) 61:2300239. doi: 10.1183/13993003.00239-2023 36858443 PMC10066569

[21] Barnes PJ . Inflammatory mechanisms in patients with chronic obstructive pulmonary disease. J Allergy Clin Immunol. (2016) 138:16–27. doi: 10.1016/j.jaci.2016.05.011 27373322

[22] Zhang Y Wang L Yan F Yang M Gao H Zeng Y . Mettl3 mediated m6A methylation involved in epithelial-mesenchymal transition by targeting SOCS3/STAT3/SNAI1 in cigarette smoking-induced COPD. Int J Chron Obstruct Pulmon Dis. (2023) 18:1007–17. doi: 10.2147/COPD.S398289 37275442 PMC10239240

[23] Mo R Li J Chen Y Ding Y . lncRNA GAS5 promotes pyroptosis in COPD by functioning as a ceRNA to regulate the miR-223-3p/NLRP3 axis. Mol Med Rep. (2022) 26:219. doi: 10.3892/mmr.2022.12735 35583006 PMC9175270

[24] Rumora L Somborac-Bačura A Hlapčić I Hulina-Tomašković A Rajković MG . Cigarette smoke and extracellular Hsp70 induce secretion of ATP and differential activation of NLRP3 inflammasome in monocytic and bronchial epithelial cells. Cytokine. (2020) 135:155220. doi: 10.1016/j.cyto.2020.155220 32736335

[25] Ni FX Wang HX Hu J Chen PS Huang HJ Chen HH . Pyroptosis-driven immune dysregulation in COPD: molecular mechanisms and therapeutic implications. Front Immunol. (2025) 16:1686175. doi: 10.3389/fimmu.2025.1686175 41476973 PMC12747962

[26] Rice GI Del Toro Duany Y Jenkinson EM Forte GM Anderson BH Ariaudo G . Gain-of-function mutations in IFIH1 cause a spectrum of human disease phenotypes associated with upregulated type I interferon signaling. Nat Genet. (2014) 46:503–9. doi: 10.1038/ng.2933 24686847 PMC4004585

[27] Hill NR Fatoba ST Oke JL Hirst JA O'Callaghan CA Lasserson DS . Global prevalence of chronic kidney disease - a systematic review and meta-analysis. PloS One. (2016) 11:e0158765. doi: 10.1371/journal.pone.0158765 27383068 PMC4934905

[28] Ruiz-Ortega M Rayego-Mateos S Lamas S Ortiz A Rodrigues-Diez RR . Targeting the progression of chronic kidney disease. Nat Rev Nephrol. (2020) 16:269–88. doi: 10.1038/s41581-019-0248-y 32060481

[29] Qian J Wang Q Xu J Liang S Zheng Q Guo X . Macrophage OTUD1-CARD9 axis drives isoproterenol-induced inflammatory heart remodelling. Clin Transl Med. (2024) 14:e1790. doi: 10.1002/ctm2.1790 39118286 PMC11310286

[30] Guo S Li F Wills M Yip J Wehbe A Peng C . Chlorpromazine and Promethazine (C+P) reduce brain injury after ischemic stroke through the PKC-δ/NOX/MnSOD pathway. Mediators Inflammation. (2022) 2022:6886752. doi: 10.1155/2022/6886752 35873710 PMC9307415

[31] Jiang Z Chen Q Yang H . Drug delivery strategies for neuroprotective therapy in ischemic stroke: application of nanotechnology. Neural Regener Res. (2025) 21(5):1793–808. doi: 10.4103/NRR.NRR-D-24-01383 40364653 PMC12694626

[32] Vongsfak J Pratchayasakul W Apaijai N Vaniyapong T Chattipakorn N Chattipakorn SC . The alterations in mitochondrial dynamics following cerebral ischemia/reperfusion injury. Antioxidants (Basel). (2021) 10:1384. doi: 10.3390/antiox10091384 34573016 PMC8468543

[33] Leech T Chattipakorn N Chattipakorn SC . The beneficial roles of metformin on the brain with cerebral ischaemia/reperfusion injury. Pharmacol Res. (2019) 146:104261. doi: 10.1016/j.phrs.2019.104261 31170502

[34] Chen S Chen H Du Q Shen J . Targeting myeloperoxidase (MPO) mediated oxidative stress and inflammation for reducing brain ischemia injury: potential application of natural compounds. Front Physiol. (2020) 11:433. doi: 10.3389/fphys.2020.00433 32508671 PMC7248223

[35] Jiang H Wang X Lei W Song P Cheng J . Allicin alleviates cerebral ischemia/reperfusion injury by inhibiting neuronal mitochondrial oxidative stress via OTUD1/NRF2 axis. Phytomedicine. (2025) 148:157468. doi: 10.1016/j.phymed.2025.157468 41172920

[36] Zhang Q Chen Z Li J Huang K Ding Z Chen B . The deubiquitinase OTUD1 stabilizes NRF2 to alleviate hepatic ischemia/reperfusion injury. Redox Biol. (2024) 75:103287. doi: 10.1016/j.redox.2024.103287 39079388 PMC11340619

[37] Wang J Ge X Lang C Xu W Hu T Wang L . Athlete-derived extracellular vesicles protect against spinal cord injury via inhibition of neuronal ferroptosis. Sci Adv. (2025) 11:eadx7695. doi: 10.1126/sciadv.adx7695 41385620 PMC12700199

[38] Shi S Zhang Z Shi P Gong C Zhao Z Wang T . Exosomal miR-494 from bone marrow mesenchymal stem cells attenuates ferroptosis and enhances spinal cord injury repair by modulating the SIRT1/HO-1 pathway. Mol Neurobiol. (2025) 62:16575–91. doi: 10.1007/s12035-025-05164-1 40913206

[39] Li D Lu X Xu G Liu S Gong Z Lu F . Dihydroorotate dehydrogenase regulates ferroptosis in neurons after spinal cord injury via the P53-ALOX15 signaling pathway. CNS Neurosci Ther. (2023) 29:1923–39. doi: 10.1111/cns.14150 36942513 PMC10324365

[40] She W Su J Ma W Ma G Li J Zhang H . Natural products protect against spinal cord injury by inhibiting ferroptosis: a literature review. Front Pharmacol. (2025) 16:1557133. doi: 10.3389/fphar.2025.1557133 40248093 PMC12003294

[41] Li D Zhu B Jiao W Chen W Zhang X Li D . Apoptotic extracellular vesicles derived from UC-MSCs promote spinal cord injury repair by inhibiting ferroptosis via OTUD1. Free Radic Biol Med. (2025) 242:120–37. doi: 10.1016/j.freeradbiomed.2025.10.270 41151199

[42] Kim EJ Kim YJ Lee HI Jeong SH Nam HJ Cho JH . NRF2 knockdown resensitizes preclinically 5-fluorouracil-resistant pancreatic cancer cells by suppressing HO-1 and ABCG2 expression. Int J Mol Sci. (2020) 21:4646. doi: 10.3390/ijms21134646 32629871 PMC7369955

[43] Mukhopadhyay S Goswami D Adiseshaiah PP Burgan W Yi M Guerin TM . Undermining glutaminolysis bolsters chemotherapy while NRF2 promotes chemoresistance in KRAS-driven pancreatic cancers. Cancer Res. (2020) 80:1630–43. doi: 10.1158/0008-5472.CAN-19-1363 31911550 PMC7185043

[44] Grattarola M Cucci MA Roetto A Dianzani C Barrera G Pizzimenti S . Post-translational down-regulation of Nrf2 and YAP proteins, by targeting deubiquitinases, reduces growth and chemoresistance in pancreatic cancer cells. Free Radic Biol Med. (2021) 174:202–10. doi: 10.1016/j.freeradbiomed.2021.08.006 34364982

[45] Chen Y Xiong J Xiao Y Li B Su Z Jiang L . OTUD1 relieves coxsackievirus-induced ferroptosis and inflammation in myocardial cells by stabilizing NRF2 and inhibiting NF-κB signaling. IUBMB Life. (2026) 78:e70094. doi: 10.1002/iub.70094 41853919

[46] Wan Q Hao J Chen C . Fluoxetine alleviates vascular cognitive impairment by activating the Nrf2/ARE pathway via Sp1-mediated OTUD1 transcription. Free Radic Res. (2026) 60(3):241–57. doi: 10.1080/10715762.2026.2658796 41969155

[47] Bae ES Byun WS Ock CW Kim WK Park HJ Lee SK . Periplocin exerts antitumor activity by regulating Nrf2-mediated signaling pathway in gemcitabine-resistant pancreatic cancer cells. BioMed Pharmacother. (2023) 157:114039. doi: 10.1016/j.biopha.2022.114039 36423542

[48] Zhang J Xu HX Cho WCS Cheuk W Li Y Huang QH . Brucein D augments the chemosensitivity of gemcitabine in pancreatic cancer via inhibiting the Nrf2 pathway. J Exp Clin Cancer Res. (2022) 41:90. doi: 10.1186/s13046-022-02270-z 35272669 PMC8908700

[49] Antonucci L Watari K Feng Y Qi J Zhu M Wang T . Selective targeting of NRF2-high pancreatic ductal adenocarcinoma with an NQO1-activatable prodrug. Proc Natl Acad Sci USA. (2026) 123:e2511733123. doi: 10.1073/pnas.2511733123 41564125 PMC12849708

[50] Lv Y Tang W Xu Y Chang W Zhang Z Lin Q . Apolipoprotein L3 enhances CD8+ T cell antitumor immunity of colorectal cancer by promoting LDHA-mediated ferroptosis. Int J Biol Sci. (2023) 19:1284–98. doi: 10.7150/ijbs.74985 36923931 PMC10008698

[51] Xie XT Pang QH Luo LX . Role of targeting ferroptosis as a component of combination therapy in combating drug resistance in colorectal cancer. World J Clin Oncol. (2024) 15:375–7. doi: 10.5306/wjco.v15.i3.375 38576594 PMC10989259

[52] Zhang Y Du J Cui X Ling Y Tang C . Development of a bispecific CDH17-GUCY2C ADC bearing the ferroptosis inducer RSL3 for the treatment of colorectal cancer. Cell Death Discov. (2025) 11:347. doi: 10.1038/s41420-025-02652-0 40721409 PMC12304242

[53] Song J Liu T Yin Y Zhao W Lin Z Yin Y . The deubiquitinase OTUD1 enhances iron transport and potentiates host antitumor immunity. EMBO Rep. (2021) 22:e51162. doi: 10.15252/embr.202051162 33393230 PMC7857436

[54] Wang M Zhang R He S Wen F Yu X Xu X . Targeting FBXL5 to induce ferroptosis and reverse oxaliplatin resistance in iron-rich colorectal cancer. Sci Rep. (2025) 15:37189. doi: 10.1038/s41598-025-14086-w 41136490 PMC12552431

[55] Lu L Huang X Zhou Y Zhao Y Zou Y Huang W . Yangjing Zhongyu decoction ameliorates polycystic ovary syndrome via multi-organ ferroptosis inhibition: A mechanistic study. J Ethnopharmacol. (2026) 361:121186. doi: 10.1016/j.jep.2026.121186 41571019

[56] Zhu F Liu X Li H Li J Liu H Wang Y . Identification of a novel ferroptosis-induced immunogenic cell death related signature based on a machine learning framework in colorectal cancer. Discov Oncol. (2025) 16:1289. doi: 10.1007/s12672-025-03147-1 40632358 PMC12240883

[57] Han Y Wang H Guo Y Zhang H Wen X Zhang M . The histone demethylase KDM4A promotes esophageal cancer progression by epigenetically activating LRG1-mediated TGF-β signaling. Apoptosis. (2026) 31:96. doi: 10.1007/s10495-026-02294-2 41811529

[58] Luo Q Wu X Zhao P Nan Y Chang W Zhu X . OTUD1 activates caspase-independent and caspase-dependent apoptosis by promoting AIF nuclear translocation and MCL1 degradation. Adv Sci (Weinh). (2021) 8:2002874. doi: 10.1002/advs.202002874 33898171 PMC8061361

[59] Siegel RL Miller KD Jemal A . Cancer statistics, 2019. CA Cancer J Clin. (2019) 69:7–34. doi: 10.3322/caac.21551 30620402

[60] Liu B Zhang J Meng X Wang S Zhang J Li B . HDAC6-G3BP2 promotes lysosomal-TSC2 and suppresses mTORC1 under ETV4 targeting-induced low-lactate stress in non-small cell lung cancer. Oncogene. (2023) 42:1181–95. doi: 10.1038/s41388-023-02641-6 36823378

[61] Cheng R Zhang G Bai Y Zhang F Zhang G . LncRNA SENCR promotes cell proliferation and progression in non-small-cell lung cancer cells via sponging miR-1-3p. Cell Cycle. (2021) 20:1402–14. doi: 10.1080/15384101.2021.1924958 34224326 PMC8344740

[62] Genova C . The long run towards personalized therapy in non-small-cell lung cancer: Current state and future directions. Int J Mol Sci. (2023) 24:8212. doi: 10.3390/ijms24098212 37175919 PMC10178998

[63] Zuo L Zou X Ge J Hu S Fang Y Xu Y . The Nrf2-HMOX1 pathway as a therapeutic target for reversing cisplatin resistance in non-small cell lung cancer via inhibiting ferroptosis. Cell Death Discov. (2025) 11:287. doi: 10.1038/s41420-025-02564-z 40544155 PMC12182566

[64] Liu H Zhong L Lu Y Liu X Wei J Ding Y . Deubiquitylase OTUD1 confers Erlotinib sensitivity in non-small cell lung cancer through inhibition of nuclear translocation of YAP1. Cell Death Discov. (2022) 8:403. doi: 10.1038/s41420-022-01119-w 36182943 PMC9526728

[65] Ma X Wang L Shi G Sun S . The deubiquitinase OTUD1 inhibits non-small cell lung cancer progression by deubiquitinating and stabilizing KLF4. Thorac Cancer. (2022) 13:761–70. doi: 10.1111/1759-7714.14320 35098684 PMC8888149

[66] Zhang Q Li J Chen Z Jiang K Yang K Huang F . VE-822 upregulates the deubiquitinase OTUD1 to stabilize FHL1 to inhibit the progression of lung adenocarcinoma. Cell Oncol (Dordr). (2023) 46:1001–14. doi: 10.1007/s13402-023-00793-x 36929488 PMC12974673

[67] Geng C Dong K An J Liu Z Zhao Q Lv Y . OTUD3 inhibits breast cancer cell metastasis by regulating TGF-β pathway through deubiquitinating SMAD7. Cancer Cell Int. (2025) 25:181. doi: 10.1186/s12935-025-03822-x 40382618 PMC12085847

[68] Won HS Ahn J Kim Y Kim JS Song JY Kim HK . Clinical significance of HER2-low expression in early breast cancer: a nationwide study from the Korean Breast Cancer Society. Breast Cancer Res. (2022) 24:22. doi: 10.1186/s13058-022-01519-x 35307014 PMC8935777

[69] Sun L Jia X Wang K Li M . Unveiling the future of breast cancer therapy: Cutting-edge antibody-drug conjugate strategies and clinical outcomes. Breast. (2024) 78:103830. doi: 10.1016/j.breast.2024.103830 39500221 PMC11570738

[70] Fu F Zhang D Hu L Sundaram S Ying D Zhang Y . Association between 15 known or potential breast cancer susceptibility genes and breast cancer risks in Chinese women. Cancer Biol Med. (2021) 19:253–62. doi: 10.20892/j.issn.2095-3941.2021.0358 34606182 PMC8832954

[71] Kurian AW Abrahamse P Bondarenko I Hamilton AS Deapen D Gomez SL . Association of genetic testing results with mortality among women with breast cancer or ovarian cancer. J Natl Cancer Inst. (2022) 114:245–53. doi: 10.1093/jnci/djab151 34373918 PMC8826508

[72] Fan C González-Prieto R Kuipers TB Vertegaal ACO van Veelen PA Mei H . The lncRNA LETS1 promotes TGF-β-induced EMT and cancer cell migration by transcriptionally activating a TβR1-stabilizing mechanism. Sci Signal. (2023) 16:eadf1947. doi: 10.1126/scisignal.adf1947 37339182

[73] Xu T Liu H Ling N Chen D Dai J Chen W . OTUD3-mediated stabilization of SLC7A11 drives sunitinib resistance by suppressing ferroptosis in clear cell renal cell carcinoma. Cancer Lett. (2025) 632:217942. doi: 10.1016/j.canlet.2025.217942 40716486

[74] TaoW DongY ZuoS WangH LiangQ CaiT . DR5/WDR12 balances p65 stability promoting sunitinib resistance in renal cell carcinoma. Cell Death Differ. (2026). doi: 10.1038/s41418-026-01723-8 41872532

[75] Li W Shi J Lv Q Miao D Tan D Lu X . Identification of the LCOR-PLCL1 pathway that restrains lipid accumulation and tumor progression in clear cell renal cell carcinoma. Int J Biol Sci. (2025) 21:2295–312. doi: 10.7150/ijbs.107981 40083699 PMC11900815

[76] Liu W Yan B Yu H Ren J Peng M Zhu L . OTUD1 stabilizes PTEN to inhibit the PI3K/AKT and TNF-alpha/NF-kappaB signaling pathways and sensitize ccRCC to TKIs. Int J Biol Sci. (2022) 18:1401–14. doi: 10.7150/ijbs.68980 35280681 PMC8898358

[77] Wisztorski M Aboulouard S Roussel L Duhamel M Saudemont P Cardon T . Fallopian tube lesions as potential precursors of early ovarian cancer: a comprehensive proteomic analysis. Cell Death Dis. (2023) 14:644. doi: 10.1038/s41419-023-06165-5 37775701 PMC10541450

[78] Chandrasekaran A Elias KM . Synthetic lethality in ovarian cancer. Mol Cancer Ther. (2021) 20:2117–28. doi: 10.1158/1535-7163.MCT-21-0500 34518297 PMC8571039

[79] Wu Z Zhuang X Zhan X Chen W Xie Y Zhou Z . STAT1 and IL-7 as potential diagnostic biomarkers for distinguishing high-grade from low-grade serous ovarian cancer: a multi-cohort analysis. Front Immunol. (2026) 17:1779912. doi: 10.3389/fimmu.2026.1779912 42058211 PMC13120972

[80] Ma H Qi G Han F Lu W Peng J Li R . HMGB3 promotes PARP inhibitor resistance through interacting with PARP1 in ovarian cancer. Cell Death Dis. (2022) 13:263. doi: 10.1038/s41419-022-04670-7 35332131 PMC8948190

[81] Chen T Ho M Briere J Moscvin M Czarnecki PG Anderson KC . Multiple myeloma cells depend on the DDI2/NRF1-mediated proteasome stress response for survival. Blood Adv. (2022) 6:429–40. doi: 10.1182/bloodadvances.2020003820 34649278 PMC8791580

[82] Zhou X He R Hu WX Luo S Hu J . Targeting myeloma metabolism: How abnormal metabolism contributes to multiple myeloma progression and resistance to proteasome inhibitors. Neoplasia. (2024) 50:100964. doi: 10.1016/j.neo.2024.100964 38364355 PMC10881428

[83] Wang Y Vandewalle N De Veirman K Vanderkerken K Menu E De Bruyne E . Targeting mTOR signaling pathways in multiple myeloma: biology and implication for therapy. Cell Commun Signal. (2024) 22:320. doi: 10.1186/s12964-024-01699-3 38862983 PMC11165851

[84] Bilski R Kupczyk D Kaczorowska-Bilska K Tkaczenko H Kurhaluk N Kosmalski T . Oxidative stress in multiple myeloma: Pathogenic mechanisms, biomarkers, and redox-targeted therapeutic strategies. Int J Mol Sci. (2026) 27:3001. doi: 10.3390/ijms27073001 41977190 PMC13073741

[85] Vdovin A Jelinek T Zihala D Sevcikova T Durech M Sahinbegovic H . The deubiquitinase OTUD1 regulates immunoglobulin production and proteasome inhibitor sensitivity in multiple myeloma. Nat Commun. (2022) 13:6820. doi: 10.1038/s41467-022-34654-2 36357400 PMC9649770

[86] Jia G Hill MA Sowers JR . Diabetic cardiomyopathy: An update of mechanisms contributing to this clinical entity. Circ Res. (2018) 122:624–38. doi: 10.1161/CIRCRESAHA.117.311586 29449364 PMC5819359

[87] Han X Zheng R Zhang J Liu Y Li Z Liu G . Cardiomyocyte OTUD1 drives diabetic cardiomyopathy via directly deubiquitinating AMPKα2 and inducing mitochondrial dysfunction. Nat Commun. (2025) 16:6668. doi: 10.1038/s41467-025-61901-z 40683882 PMC12276362

[88] Hausenloy DJ Yellon DM . Myocardial ischemia-reperfusion injury: a neglected therapeutic target. J Clin Invest. (2013) 123:92–100. doi: 10.1172/JCI62874 23281415 PMC3533275

[89] Luo Y Li WX Zheng QS Yan JQ Yang YD Shen SR . OTUD1 deficiency attenuates myocardial ischemia/reperfusion induced cardiomyocyte apoptosis by regulating RACK1 phosphorylation. Acta Pharmacol Sin. (2025) 46:2649–62. doi: 10.1038/s41401-025-01567-x 40394237 PMC12460603

[90] Preston KJ Kawai T Torimoto K Kuroda R Nakayama Y Akiyama T . Mitochondrial fission inhibition protects against hypertension induced by angiotensin II. Hypertens Res. (2024) 47:1338–49. doi: 10.1038/s41440-024-01610-0 38383894

[91] Takayanagi T Forrester SJ Kawai T Obama T Tsuji T Elliott KJ . Vascular ADAM17 as a novel therapeutic target in mediating cardiovascular hypertrophy and perivascular fibrosis induced by angiotensin II. Hypertension. (2016) 68:949–55. doi: 10.1161/HYPERTENSIONAHA.116.07620 27480833 PMC5016240

[92] Shang P Liu T Liu W Li Y Dou F Zhang Y . Telmisartan improves vascular remodeling through ameliorating prooxidant and profibrotic mechanisms in hypertension via the involvement of transforming growth factor-β1. Mol Med Rep. (2017) 16:4537–44. doi: 10.3892/mmr.2017.7177 28791353 PMC5646990

[93] Zhou X Xing Y Wang Y Lv M Zhang P Zhu S . OTUD1 regulates cytokine expression and related pathways in goose fatty liver by promoting deubiquitination of its target proteins. Poult Sci. (2024) 103:104382. doi: 10.1016/j.psj.2024.104382 39437555 PMC11532766

[94] Liu Q Mallette E Zheng H Zhang W . Development of an OTUD1 ubiquitin variant inhibitor. Biochem J. (2023) 480:1317–30. doi: 10.1042/BCJ20230119 37589489

[95] Woo SM Seo SU Min KJ Kwon TK . Melatonin induces apoptotic cell death through Bim stabilization by Sp1-mediated OTUD1 upregulation. J Pineal Res. (2022) 72:e12781. doi: 10.1111/jpi.12781 34826170

